# Comparing Human Ability with Generative AI: Self-Evaluation, Appraisal, and Adaptive Professional Intelligence Among Journalism and Communication Students

**DOI:** 10.3390/jintelligence14090201

**Published:** 2026-09-01

**Authors:** Juan Wang, Zichen Liu, Jiaying Huang

**Affiliations:** 1 Faculty of International Media, Communication University of China, Beijing 100024, China; seven0246813579@163.com; 2 School of Humanities, Communication University of China, Beijing 100024, China; 18931024671@163.com

**Keywords:** human–AI comparison, perceived professional value, job replacement anxiety, AI learning motivation, AI appraisal, journalism and communication education

## Abstract

Generative artificial intelligence (AI) is reshaping how students evaluate their abilities, occupational prospects, and the continuing value of professional education. This study examined how ability-based comparison with AI is associated with perceived professional value among journalism and communication students and why the same comparison may be linked to both threatening and adaptive responses. An explanatory sequential mixed-methods design was used. Survey data from 507 students in China were analyzed using partial least squares structural equation modeling, with PROCESS analyses as robustness checks, followed by semi-structured interviews with 20 students. Ability-based comparison with AI was positively associated with job replacement anxiety, which was negatively associated with perceived professional value, and with AI learning motivation, which was positively associated with perceived professional value. A positive direct association with perceived professional value also remained. AI hindrance appraisal strengthened the comparison–anxiety association, whereas AI challenge appraisal did not significantly moderate the comparison–motivation association. Interviews indicated that these relationships were task- and criterion-dependent and shaped by occupational interpretation and concrete learning demands. Human–AI comparison may therefore relate to professional value through simultaneous threat- and adaptation-oriented pathways rather than a uniformly positive or negative process.

## 1. Introduction

The rapid development of generative artificial intelligence (AI) is reshaping the relationship between humans and knowledge work. Unlike earlier digital technologies that primarily facilitated information access and communication, generative AI systems can directly participate in cognitive and creative tasks, including writing, summarization, translation, information synthesis, and content generation (Dwivedi et al., 2023; Kasneci et al., 2023). In higher education, these systems can support personalized learning, rapid feedback, and knowledge production, but they may also create concerns about overreliance, reduced cognitive engagement, and the continuing relevance of human expertise (Abbas et al., 2024; Chan & Hu, 2023; Giannakos et al., 2025; Kasneci et al., 2023; L. Yan et al., 2024). As AI-generated outputs become increasingly comparable with human-produced work, students may begin to reconsider not only how they use AI, but also how they evaluate their own abilities and the value of their professional training.

This issue is particularly salient in journalism and communication education. Generative AI is increasingly used in news writing, editing, translation, information retrieval, data analysis, and multimedia production (Cools & Diakopoulos, 2026). Many of these activities are central to students’ coursework, internships, and preparation for entry-level employment (Aitamurto & Boyles, 2025; Cools & Diakopoulos, 2026). However, journalism and communication competence cannot be reduced to the efficient production of content. It also involves source verification, contextual interpretation, ethical judgment, public accountability, and responsibility for the social consequences of communication (Cools & de Vreese, 2025; Dierickx et al., 2024). Although AI systems can generate plausible content and assist professional decision-making, responsibility for accuracy, fairness, harm, and the public interest remains attached to human professionals and institutions (de-Lima-Santos et al., 2025; Dodds et al., 2025; Paik, 2025; Porlezza & Schapals, 2024). Journalism students therefore encounter a tension between the growing technical capabilities of AI and the continuing importance of professional judgment. Consistent with this tension, recent studies show that journalism students and practitioners recognize AI’s potential to improve productivity while also expressing concerns about accuracy, authenticity, transparency, professional autonomy, and journalistic norms (Aitamurto & Boyles, 2025; Cools & Diakopoulos, 2026).

China provides a relevant context for examining this issue. Recent national policies have promoted the integration of AI into university curricula, teaching practices, interdisciplinary training, and students’ employment-related capabilities (Ministry of Education of the People’s Republic of China et al., 2025). Research on Chinese higher education similarly indicates that AI is being institutionalized through policy direction, university-level experimentation, general AI education, and discipline-specific applications, while concerns remain about overreliance, academic integrity, and the appropriate role of human judgment (X. Liu et al., 2025). Within journalism, recent evidence also suggests that Chinese young journalists’ engagement with AI is shaped not only by technological utility but also by professional norms and understandings of journalistic work (Y. Liu, 2025). For Chinese journalism and communication students, AI is therefore becoming both an emerging component of professional competence and a source of uncertainty about future skill requirements and employment.

Existing research has offered two contrasting accounts of these developments. Studies of generative AI in higher education have emphasized its capacity to improve learning efficiency, provide academic support, and expand students’ access to knowledge and new forms of skill development (Chan & Hu, 2023; Crompton & Burke, 2023; Tlili et al., 2023). In contrast, research on automation and technological change has highlighted concerns about job insecurity, occupational displacement, professional autonomy, and the declining relevance of established skills (Brougham & Haar, 2018; Frey & Osborne, 2017; Kim & Kim, 2024; Shoss, 2017). These perspectives explain why AI may be experienced as either an enabling resource or a professional threat, but they have largely developed along separate lines. Consequently, less is known about the psychological process through which individuals interpret perceived differences between their own abilities and AI capabilities, or why the same comparison experience may be associated with both anxiety and motivation.

Human–AI ability comparison provides a useful starting point for addressing this issue. When students observe AI performing tasks related to their professional training, those outputs may become relevant information for evaluating their own competence. When perceived capability differences are interpreted as signals of diminished future professional value, ability-based comparison with AI may be associated with stronger job replacement anxiety; when comparison information is understood as specifying concrete and addressable capability requirements, it may also be associated with stronger AI learning motivation.

Despite increasing attention to generative AI, three questions remain insufficiently addressed. First, existing studies have focused primarily on AI use, acceptance, and general attitudes, with less attention to students’ comparison of their own professional abilities with AI performance. Second, empowering and threatening responses to AI have often been examined separately, leaving unclear why the same tendency to engage in ability-based comparison may be simultaneously associated with psychologically divergent responses. Third, limited research has examined how these responses are related to students’ perceived professional value or whether the strength of these associations varies according to students’ appraisal of AI. These gaps are especially relevant in professional fields such as journalism and communication, where technological capability and human judgment increasingly coexist.

To address these questions, the present study examines how ability-based comparison with generative AI relates to journalism and communication students’ perceived professional value. Drawing on Social Comparison Theory and the Transactional Model of Stress and Coping, it develops a model involving job replacement anxiety, AI learning motivation, and challenge and hindrance appraisals of AI. In doing so, the study seeks to connect previously separate accounts of AI-related opportunity and professional uncertainty and to examine two possible psychological pathways through which ability-based comparison with AI is associated with perceived professional value. Using an explanatory sequential mixed-methods design, the study first tests the proposed relationships with survey data from 507 journalism and communication students in China and subsequently uses semi-structured interviews with 20 students to provide contextual and process-oriented explanations of the quantitative findings.

## 2. Literature Review and Hypotheses

### 2.1. Ability-Based Comparison with AI and Professional Self-Evaluation

Social Comparison Theory begins with the proposition that individuals possess a fundamental need to evaluate their abilities and opinions (Festinger, 1954). When objective criteria are unavailable or insufficiently clear, people obtain evaluative information by comparing themselves with relevant referents. Such comparison is not simply the observation of another performer. It is a relational judgment in which individuals locate their own ability relative to an external standard and use the perceived discrepancy to infer what they can currently accomplish, where they stand, and whether improvement is needed. Later developments of the theory have further shown that comparison is shaped by the relevance of the comparison dimension, the perceived diagnostic value of the target, and the implications of the observed difference for the self (Buunk & Gibbons, 2007; Suls et al., 2002).

Comparison direction is one of the central mechanisms of this process. Individuals may engage in upward comparison with a target perceived as performing better, downward comparison with a target perceived as performing worse, or lateral comparison with a target perceived as similar (Gerber et al., 2018). Upward comparison is especially consequential because it supplies information about a higher level of performance, but its psychological consequences are not uniform. Upward comparison may produce adverse self-evaluation when individuals focus on their relative disadvantage (Collins, 1996; Gerber et al., 2018). It may also encourage constructive self-improvement when the superior performance is relevant to personal goals and appears informative or potentially attainable (Lockwood & Kunda, 1997).

One possible outcome is a contrast effect. Contrast occurs when individuals focus on the distance separating their current ability from a superior level of performance, causing their own competence to appear comparatively weak (Collins, 1996). This response is particularly likely when the comparison dimension is important to the self or the discrepancy appears difficult to close (Gerber et al., 2018). A second possible outcome is an assimilation or self-improvement effect. In this case, superior performance is treated as informative and relevant to one’s own development. Rather than merely confirming inadequacy, the observed difference indicates a possible future state or identifies strategies and competencies that can be acquired (Lockwood & Kunda, 1997). Comparison outcomes may also depend on whether individuals selectively focus on differences from or similarities with the comparison target, with the former facilitating contrast and the latter facilitating assimilation (Mussweiler, 2003).

These mechanisms explain why the same comparison experience can generate different psychological responses. Contrast emphasizes distance, relative disadvantage, and possible loss of competence (Collins, 1996), whereas assimilation emphasizes useful information, attainable development, and movement toward a higher standard (Lockwood & Kunda, 1997). Whether comparison leads primarily to one response or the other depends on how individuals understand the comparison target’s relevance, the perceived attainability of its performance, and the implications of the observed difference for their goals (Buunk & Gibbons, 2007; Wood, 1989).

Digital technologies have expanded the range of referents from which individuals obtain such self-evaluative information. Research on technology-mediated comparison has shown that visible information about performance and achievement can influence self-evaluation even when comparison occurs indirectly through a digital environment (Vogel et al., 2014). More recent research demonstrates that individuals may also compare their performance with technological agents. Social comparison with workplace robots has been associated with stronger perceived job insecurity (P. X. Wang et al., 2023). In the context of generative AI, researchers have distinguished ability-based comparison from opinion-based comparison and found that ability-based comparison is associated with identity threat, while both forms of comparison are also positively related to self-esteem (Huang et al., 2025).

Generative AI makes ability comparison particularly immediate because users can place their own work alongside AI-generated outputs. For students, they can compare their writing, translations, summaries, analyses, or creative ideas with outputs produced by AI and evaluate differences in speed, linguistic fluency, informational scope, organization, or apparent quality. In the present study, ability-based comparison with AI (ASC) refers to the extent to which students attend to and evaluate differences between their own professional abilities and the performance displayed by generative AI. This conceptualization draws on the ability-comparison dimension of social comparison orientation and extends it to interactions with generative AI (Gibbons & Buunk, 1999; Huang et al., 2025). When students perceive AI as performing better on a professionally relevant task, the contrast and assimilation mechanisms provide two theoretical routes through which comparison may acquire personal and professional meaning (Collins, 1996; Lockwood & Kunda, 1997).

This process is especially relevant in journalism and communication because the field contains both readily comparable production tasks and forms of expertise that are less easily evaluated through an isolated output. Recent occupational research suggests that AI may automate some tasks while enhancing productivity, augmenting professional decision-making, or creating demand for AI-enabling forms of expertise in other areas (Q. Wang et al., 2024). Generative AI can assist with drafting, editing, transcription, translation, summarization, information organization, and content adaptation (Cools & Diakopoulos, 2026). Journalism and communication work, however, also require source verification, field-based information gathering, contextual interpretation, ethical judgment, and accountability for the consequences of publication. Journalists recognize the efficiency and data-processing potential of generative AI while remaining concerned about accuracy, transparency, professional autonomy, and journalistic norms (Cools & Diakopoulos, 2026). Journalism students similarly combine expectations of productivity and innovation with concerns about authenticity, trust, transparency, and journalism’s public service mission (Aitamurto & Boyles, 2025).

Consequently, comparison with AI may prompt students to evaluate more than their performance on an individual assignment. It may lead them to reconsider which competencies remain distinctive, which professional tasks are likely to change, and whether their training continues to prepare them for socially and occupationally meaningful work.

An important outcome of this process is perceived professional value (PPV), which refers to students’ judgments about whether their professional field, training, knowledge, and prospective work remain socially meaningful, professionally relevant, and worth continued investment (Pratt et al., 2006; Trede et al., 2012). Understanding PPV is important because students’ beliefs about the continuing significance of their training may shape their professional learning, career preparation, and responses to technological change (Chan & Hu, 2023; Tomlinson & Jackson, 2021; Voigt & Strauss, 2024). The current manuscript similarly treats PPV as an evaluation of the field and its training rather than as a direct measure of professional identity. PPV is an evaluative judgment concerning the professional field, professional training, and future professional work; it does not indicate whether students identify themselves as members of the profession, nor is it equivalent to intentions to continue their studies, occupational choice, or actual educational returns.

Social Comparison Theory does not support a single directional prediction regarding the direct relationship between ASC and PPV. In contrast, the perceived superiority of AI may make students’ own abilities and training appear less distinctive, thereby weakening PPV (Collins, 1996). Through assimilation, the same comparison may make new developmental requirements more salient and support a more adaptive interpretation of professional change (Lockwood & Kunda, 1997). Comparison may also make the continuing significance of human judgment more visible. Recent human–AI comparison research likewise suggests that ability comparison can be associated with divergent self-evaluative responses rather than a uniformly negative or positive outcome (Huang et al., 2025).

The study therefore poses the following research question:

Research Question 1 (RQ1): How is ability-based comparison with AI associated with journalism and communication students’ perceived professional value?

### 2.2. Threat-Based Pathway: Job Replacement Anxiety

The first proposed pathway follows the contrast mechanism of social comparison. When attention is directed toward a superior performance standard, individuals may experience their own competence as comparatively deficient (Collins, 1996). This response is particularly likely when the comparison dimension is personally important and the observed difference appears difficult to overcome (Gerber et al., 2018). In professional education, the implications of contrast can extend beyond current performance because students may interpret a capability gap as evidence about their future employability and the continuing demand for human expertise (Wood, 1989).

This inference connects social comparison with research on technological job insecurity. Job insecurity is not determined solely by actual job loss; it also arises from the subjective anticipation that employment, valued tasks, or occupational opportunities may become less secure (Shoss, 2017). Technological change can therefore generate insecurity before organizations eliminate positions because individuals anticipate changes in labor demand, skill requirements, and the economic value assigned to human work (Brougham & Haar, 2018). Time-lagged research has also found that organizational AI adoption is associated with lower self-perceived employability, although subsequent career responses depend on how individuals envision their future work selves (Lin et al., 2024).

Social comparison provides a mechanism through which technological capability may become a personally meaningful employment threat. Across seven studies, P. X. Wang et al. (2023) found that employees’ comparison with robots helped explain why humanlike robotic systems increased perceived job insecurity. This evidence is relevant because it demonstrates that technological displacement concerns do not arise only from abstract forecasts about automation. They can also emerge when individuals evaluate their own performance and occupational role relative to a technological agent capable of completing similar tasks.

In the present study, job replacement anxiety (JRA) refers to students’ concern that generative AI may reduce future opportunities for human professionals, replace selected occupational roles, weaken demand for existing professional competencies, or make entry-level employment less secure. It is an anticipatory psychological response rather than an estimate of the actual probability that specific journalism and communication positions will disappear. This distinction is consistent with research treating job insecurity as a subjective anticipation of employment-related threat (Shoss, 2017) and AI-induced job insecurity as a psychological response to perceived technological substitution (Kim & Kim, 2024).

Comparison makes AI-related capability differences more personally salient. Comparison with technological agents has been associated with perceived job insecurity (P. X. Wang et al., 2023), while organizational AI adoption has been associated with lower self-perceived employability (Lin et al., 2024). When students observe that AI can rapidly perform tasks associated with their studies and prospective occupations, they may interpret their relative disadvantage as evidence that employers will need fewer human workers or place less value on conventional training. The comparison therefore shifts the issue from “AI can perform this task” to “the ability I am developing may no longer provide professional security.” ASC captures the tendency to engage in ability-based comparison rather than the direction of a specific comparison outcome. The expectation in H1 therefore rests on an average tendency: students who compare themselves with AI more frequently may be more likely to encounter instances in which AI appears relatively advantaged on standardized or professionally relevant tasks and, when such differences are interpreted as relevant to future work, to report stronger job replacement anxiety. This expectation does not imply that students necessarily perceive AI as superior across all tasks or comparison episodes.

Journalism and communication students may be particularly susceptible to this inference because many visible AI capabilities overlap with tasks commonly associated with coursework and professional practice. Generative AI is already used for drafting, rewriting, transcription, translation, summarization, and content adaptation across stages of news production (Cools & Diakopoulos, 2026). Although these tasks do not represent the whole of journalism and communication work, students may extend a task-level comparison into a broader judgment about whether established production skills will continue to be valued. Journalism students’ concerns about AI’s implications for authenticity, trust, professional standards, and journalism’s future further illustrate the occupational meaning that may be attached to these technological changes (Aitamurto & Boyles, 2025).

Accordingly:

**Hypothesis 1 (H1).**  *Ability-based comparison with AI is positively associated with journalism and communication students’ job replacement anxiety.*

At the same time, perceived job insecurity is related to diminished occupational well-being, less favorable work attitudes, and uncertainty about the continuing viability of one’s employment (Shoss, 2017). For students, the threatened resource is not only a future position but also the expected return on their investment in professional education (Chan & Hu, 2023; Tomlinson & Jackson, 2021). If they anticipate that AI will assume important occupational functions, they may question whether their field and training retain lasting professional relevance (Duan et al., 2026).

Recent research illustrates several consequences of AI-related career uncertainty. Organizational AI adoption has been associated with reduced self-perceived employability and conditional changes in proactive career behavior (Lin et al., 2024). AI-induced job insecurity has also been associated with lower psychological safety and greater knowledge-hiding behavior (Kim & Kim, 2024). In addition, perceptions that AI may dominate future work have been linked to career insecurity and skill-development responses, which subsequently relate to career adaptability (Burhan, 2025). Although these studies concern employees rather than students, they indicate that perceived technological displacement can alter how individuals evaluate their capacity to build and sustain a future within a profession.

For journalism and communication students, stronger JRA may therefore weaken PPV by suggesting that the knowledge and skills acquired through professional education will no longer be sufficiently required or rewarded. Professional education shapes students’ understandings of future participation and the contribution they may make through professional practice (Trede et al., 2012). Anticipated replacement may consequently reduce confidence that journalism and communication training offer a viable and meaningful basis for future work. This reasoning does not equate employment opportunities with professional value; rather, it assumes that students may use anticipated employment opportunities and future skill demand as one source of information when judging whether their professional training remains occupationally meaningful.

Therefore:

**Hypothesis 2 (H2).**  *Job replacement anxiety is negatively associated with journalism and communication students’ perceived professional value.*

Together, H1 and H2 specify the threat-based pathway. Theoretically, stronger ASC may be accompanied by more salient contrast-oriented evaluations of professional capability and may be associated with lower perceived professional value through higher anticipatory job replacement anxiety.

### 2.3. Adaptive Pathway: AI Learning Motivation

Social comparison can also generate an adaptive response. Upward comparison is more likely to promote self-improvement when the comparison target is relevant to the individual’s goals, when the higher performance supplies useful information, and when improvement appears possible (Lockwood & Kunda, 1997). Under these conditions, individuals do not focus exclusively on their current disadvantage. They use the superior standard to identify a desired future state and to determine what changes in knowledge, strategy, or effort may be required to approach it (Collins, 1996).

Assimilation does not require individuals to believe that they can immediately reproduce the target’s performance. It requires the comparison to be interpreted as informative for development. The superior target may make new performance standards visible, provide examples of effective strategies, or clarify the competencies necessary for future achievement (Lockwood & Kunda, 1997). Comparison therefore functions as a source of self-regulatory information that connects present ability with a possible future capability (Taylor et al., 1996; Wood, 1989).

Generative AI is especially relevant to this mechanism because students can interact with rather than merely observe the comparison target. They can test prompts, request explanations, inspect revisions, compare alternatives, and evaluate why different outputs succeed or fail. These interactions can turn a perceived capability difference into information about methods, knowledge gaps, and emerging professional requirements. Voigt and Strauss (2024) found that interaction with AI was associated with greater perceived control over the future work self and more proactive career behavior when future work self-salience was high. Their findings indicate that AI interaction can support adaptive career responses when individuals possess a sufficiently clear future-oriented framework.

The adaptive meaning of comparison is also consistent with research on AI literacy. AI literacy extends beyond basic tool operation to include understanding how AI functions, recognizing its capabilities and limitations, critically evaluating outputs, communicating effectively with AI systems, and considering ethical and social consequences (D. Long & Magerko, 2020). It also encompasses knowing and understanding AI, using and applying it, evaluating and creating with it, and addressing its ethical implications (Ng et al., 2021). In professional education, an observed performance gap may therefore signal not only that students must outperform AI in every task, but also that they need to learn how to direct, assess, verify, and appropriately integrate AI-supported work.

In the present study, AI learning motivation (ALM) refers to students’ willingness and intention to acquire knowledge and skills that enable them to understand, evaluate, and use generative AI in journalism- and communication-related tasks. ALM is not equivalent to a generally positive attitude toward AI. Students may remain concerned about accuracy, ethics, or employment while still recognizing that AI-related competence has become important to their professional development. Research on educational AI adoption similarly distinguishes learning motivation from general attitudes and behavioral intentions toward technology (Şimşek et al., 2025).

Recent occupational studies support the proposition that AI-related change can stimulate capability-oriented development. Perceptions that AI may dominate future work have been associated with both career insecurity and skill-development responses, with skill development contributing to career adaptability (Burhan, 2025). Interaction with AI has also been associated with greater perceived control and proactive career behavior when future work self-salience is high (Voigt & Strauss, 2024). At the occupational level, AI may create automation risks while also enhancing productivity, augmenting professional intelligence, and enabling new forms of work (Q. Wang et al., 2024). ASC may make discrepancies between students’ current competencies and changing professional requirements more salient. By comparing their own work with AI output, students may identify abilities they need to acquire, including prompt design, output verification, data interpretation, multimodal production, and critical assessment of AI-generated information (D. Long & Magerko, 2020; Ng et al., 2021). Under an assimilation or self-improvement orientation, the capability gap becomes a developmental signal rather than solely evidence of inadequacy (Collins, 1996; Lockwood & Kunda, 1997). ASC captures the frequency and salience of ability-based comparison rather than guaranteeing that each comparison reveals a human disadvantage. The positive association proposed in H3 therefore depends on whether comparison information is perceived as diagnostic and actionable. Even when students do not regard themselves as inferior to AI on particular tasks, comparison may still help them identify emerging tools, evaluative standards, or human–AI collaboration requirements that are relevant to future professional development. In this sense, ALM refers to students’ willingness and intention to acquire AI-related knowledge and skills, rather than to the actual acquisition of such capabilities.

Accordingly:

**Hypothesis 3 (H3).**  *Ability-based comparison with AI is positively associated with journalism and communication students’ AI learning motivation.*

Motivation to acquire relevant competencies may allow students to understand technological change as something to which their professional field can respond. Self-Determination Theory suggests that motivation and competence development contribute to sustained engagement and meaningful investment in learning (Ryan & Deci, 2020). Rather than viewing existing training as a fixed stock of skills, students may regard it as a domain foundation that can be combined with new technological knowledge.

Recent educational research indicates that AI-enhanced learning can be associated with stronger intrinsic motivation and more positive learning experiences (Mohamed et al., 2025). Students’ own AI competence also shapes how they evaluate the learning and assessment value of generative AI tools (Heil et al., 2025). These findings suggest that the capacity and willingness to understand AI influence whether it is experienced as supportive of learning and development.

For journalism and communication students, the relationship between ALM and PPV does not imply that professional value is reduced to technical proficiency. AI-related learning becomes professionally meaningful when it includes the critical evaluation of AI output, recognition of technological limitations, and ethical awareness (D. Long & Magerko, 2020; Ng et al., 2021). Journalism students’ concerns about accuracy, trust, transparency, and the public service mission of journalism further suggest that adaptation must remain connected to domain-specific responsibilities (Aitamurto & Boyles, 2025). By developing the capacity to integrate AI with source verification, contextual interpretation, ethical use, audience analysis, creative judgment, and accountability, students may view journalism and communication as capable of adaptation rather than as a field whose expertise is being eliminated.

Therefore:

**Hypothesis 4 (H4).**  *AI learning motivation is positively associated with journalism and communication students’ perceived professional value.*

H3 and H4 together specify the adaptive pathway. Theoretically, under an assimilation or self-improvement orientation, stronger ASC may make higher performance standards and emerging capability requirements more salient and may be associated with higher perceived professional value through stronger AI learning motivation.

### 2.4. AI Appraisal as a Boundary Condition

The dual-pathway model explains how comparison may produce contrasting responses, but it does not yet explain why the same comparison information is more strongly associated with anxiety for some students and learning motivation for others. Social Comparison Theory focuses on how individuals evaluate themselves relative to an external standard. To explain how that standard acquires the meaning of either a professional threat or a developmental demand, the present study draws on the Transactional Model of Stress and Coping (Lazarus & Folkman, 1984).

The Transactional Model conceptualizes stress not as an inherent property of an external event, but as the result of a transaction between the person and the environment (Lazarus & Folkman, 1984). An event produces psychological consequences when individuals evaluate it as relevant to their goals, values, or well-being. The model therefore distinguishes the presence of an external demand from the cognitive appraisal through which the individual determines what that demand means and whether it requires a response (Lazarus & Folkman, 1984).

The first mechanism is primary appraisal. During primary appraisal, individuals assess whether an encounter is irrelevant, benign-positive, harmful, threatening, or challenging. Harm or loss concerns damage that has already occurred; threat concerns anticipated damage or loss; and challenge concerns the possibility of mastery, development, or gain through successfully addressing a demanding situation (Lazarus & Folkman, 1984). Because these evaluations depend on the meaning individuals assign to an encounter, objectively similar demands may produce different psychological outcomes (Webster et al., 2011).

The second mechanism is secondary appraisal. Individuals evaluate what can be done about the encounter, including their available coping options, resources, control, and ability to alter either the situation or their response to it. Primary and secondary appraisal are analytically distinguishable but interdependent: an encounter is more likely to be understood as threatening when important goals are at risk and coping resources appear insufficient, whereas it is more likely to be understood as challenging when demands are substantial but effective coping and development remain possible (Lazarus & Folkman, 1984).

Appraisal is also dynamic rather than a single, irreversible judgment. As individuals obtain new information, attempt coping strategies, and observe changes in the environment, they may reappraise the encounter (Lazarus & Folkman, 1984). This iterative process is especially relevant to technological change because users repeatedly encounter new AI capabilities, receive instruction, gain practical experience, and revise their judgments about what AI means for their competence and future work.

Applied to the present study, ASC supplies self-relevant information about a capability difference between students and generative AI. However, the existence of a perceived difference does not by itself determine whether the student becomes anxious or motivated. The student must interpret whether the difference threatens valued professional goals, creates barriers to development, identifies an opportunity for mastery, or can be addressed through new learning. The Transactional Model therefore supplements Social Comparison Theory by explaining how comparison information is assigned emotional and motivational meaning (Lazarus & Folkman, 1984).

Recent research provides support for applying this appraisal process to AI-related change. Cheng et al. (2023) found that organizational AI adoption generated challenge and hindrance appraisals that were associated with promotion-focused and prevention-focused forms of job crafting, respectively. Huang and Gursoy (2024) similarly found that challenge appraisal of AI integration was associated with greater thriving and proactive service behavior, whereas hindrance appraisal was associated with job insecurity and weaker proactive service behavior. These findings indicate that the psychological consequences of AI depend partly on the meaning individuals assign to AI-related demands.

In the present study, AI hindrance appraisal (AHA) refers to perceiving generative AI as creating constraints, uncertainty, and obstacles to professional development, whereas AI challenge appraisal (ACA) refers to perceiving AI-related demands as opportunities for learning, competence development, and future achievement (Cavanaugh et al., 2000; Searle & Auton, 2015). The application of these appraisal categories to AI-related change is supported by recent organizational research (Cheng et al., 2023). The two appraisals are modeled as boundary conditions because they indicate how students generally interpret the professional meaning of AI-related demands and, consequently, may condition the associations between the capability differences made salient through ASC and the two psychological responses examined in the model. ACA and AHA are modeled as moderators rather than mediators because they capture students’ general interpretive orientations toward AI-related demands rather than event-specific appraisals formed as a consequence of a particular comparison episode. The theoretical expectation is therefore that these interpretive orientations alter the strength of the associations between ASC and JRA or ALM, rather than constituting intervening mechanisms through which ASC operates. Because ASC, appraisal, JRA, and ALM were measured concurrently, however, the present study does not claim to establish the temporal ordering implied by this theoretical specification. JRA and ALM, by contrast, represent the anxiety-related and motivation-related responses associated with the two proposed pathways.

#### 2.4.1. AI Hindrance Appraisal and the Threat-Based Pathway

Hindrance appraisal occurs when individuals evaluate a demand as obstructing goal attainment, reducing control, or consuming resources without providing a clear route to valued gains (Cavanaugh et al., 2000). Such an appraisal directs attention toward constraints and anticipated losses and is generally associated with strain, withdrawal, and unfavorable behavioral responses (LePine et al., 2005; N. P. Podsakoff et al., 2007).

Within the Transactional Model, the effect of hindrance appraisal can be understood through the interaction between primary and secondary appraisal. At the primary level, AI-related change is judged to threaten professional development or employment goals. At the secondary level, students may perceive that they have limited influence over the speed of technological change, employer decisions, or the changing value of established skills. A capability gap revealed by ASC is therefore more likely to be interpreted as difficult to control or overcome (Lazarus & Folkman, 1984).

This mechanism explains why ASC should have a stronger relationship with JRA under high AHA. Students may notice that AI performs a task more quickly without automatically inferring that their future occupation is threatened. When AI is already appraised as an obstacle to professional development, however, the same task-level discrepancy provides evidence consistent with anticipated replacement. Hindrance appraisal of AI adoption gives technological capability an obstructive professional meaning (Cheng et al., 2023; Huang & Gursoy, 2024), while comparison with technological agents can make job insecurity personally salient (P. X. Wang et al., 2023). The comparison gap is therefore more readily extended from “AI performs this task well” to “my professional skills and opportunities are becoming less secure.”

Recent AI research provides additional evidence regarding the consequences of this threat-oriented interpretation. Hindrance appraisal of AI integration has been associated with stronger job insecurity and weaker proactive behavior (Huang & Gursoy, 2024). AI-induced job insecurity has also been associated with reduced psychological safety and greater knowledge-hiding behavior, although AI learning self-efficacy can mitigate some of these negative consequences (Kim & Kim, 2024).

Accordingly:

**Hypothesis 5 (H5).**  *AI hindrance appraisal strengthens the positive relationship between ability-based comparison with AI and job replacement anxiety.*

#### 2.4.2. AI Challenge Appraisal and the Adaptive Pathway

Challenge appraisal occurs when a demanding encounter is evaluated as containing possibilities for mastery, learning, achievement, or future gain (Lazarus & Folkman, 1984). A challenge is not necessarily easy or free from pressure. Rather, it is a condition in which the individual recognizes substantial demands while also perceiving that active coping may generate meaningful development (Cavanaugh et al., 2000). Empirical research further shows that the appraisal of a demand as challenging helps explain why demanding conditions may be associated with more favorable motivational and behavioral outcomes (Webster et al., 2011).

Primary and secondary appraisal again provide the underlying mechanism. At the primary level, AI-related change is judged as relevant to students’ professional goals and as offering possible gains in competence. At the secondary level, students consider learning, practice, experimentation, and skill acquisition as available coping responses. The capability difference identified through ASC can consequently be understood not only as a current disadvantage, but also as information about what must be learned to meet a changing professional standard (Lazarus & Folkman, 1984).

ACA should therefore strengthen the relationship between ASC and ALM. When students interpret AI-related demands as developmental opportunities, differences between their own performance and AI output are more likely to identify attainable learning objectives. The assimilation mechanism explains why a higher performance standard can become informative for self-improvement (Lockwood & Kunda, 1997). AI literacy research identifies the relevant developmental capabilities as including the operation of AI systems, critical evaluation of their outputs, recognition of their limitations, and ethically appropriate application (D. Long & Magerko, 2020; Ng et al., 2021).

Recent empirical studies provide support for this process. Challenge appraisal of organizational AI adoption has been associated with promotion-focused job crafting (Cheng et al., 2023). Challenge appraisal of AI integration has also been associated with greater thriving and proactive service behavior (Huang & Gursoy, 2024). In addition, interaction with AI has been linked to stronger perceived control over the future work self and greater proactive career behavior when future work self-salience is high (Voigt & Strauss, 2024).

Although ACA and ALM are conceptually distinct, challenge appraisal should make comparison information more likely to be used for learning. ACA concerns the meaning assigned to AI-related demands, whereas ALM concerns the willingness and intention to invest effort in acquiring AI-related competence. Challenge appraisal is therefore an interpretive orientation rather than learning motivation itself (Cavanaugh et al., 2000; Searle & Auton, 2015). When capability differences are interpreted as opportunities for mastery, the positive relationship between ASC and ALM should become stronger.

Accordingly:

**Hypothesis 6 (H6).**  *AI challenge appraisal strengthens the positive relationship between ability-based comparison with AI and AI learning motivation.*

Together, H5 and H6 integrate the two theoretical perspectives. Social Comparison Theory explains how AI performance becomes self-relevant ability information and why comparison may produce contrast or assimilation processes (Collins, 1996; Lockwood & Kunda, 1997). The Transactional Model of Stress and Coping explains how students’ appraisal of AI-related demands changes the extent to which comparison information is translated into replacement anxiety or learning motivation (Lazarus & Folkman, 1984).

These propositions address the second research question:

RQ2: Under what conditions are the associations between ability-based comparison with AI and threatening or adaptive psychological responses stronger?

**Figure 1:**
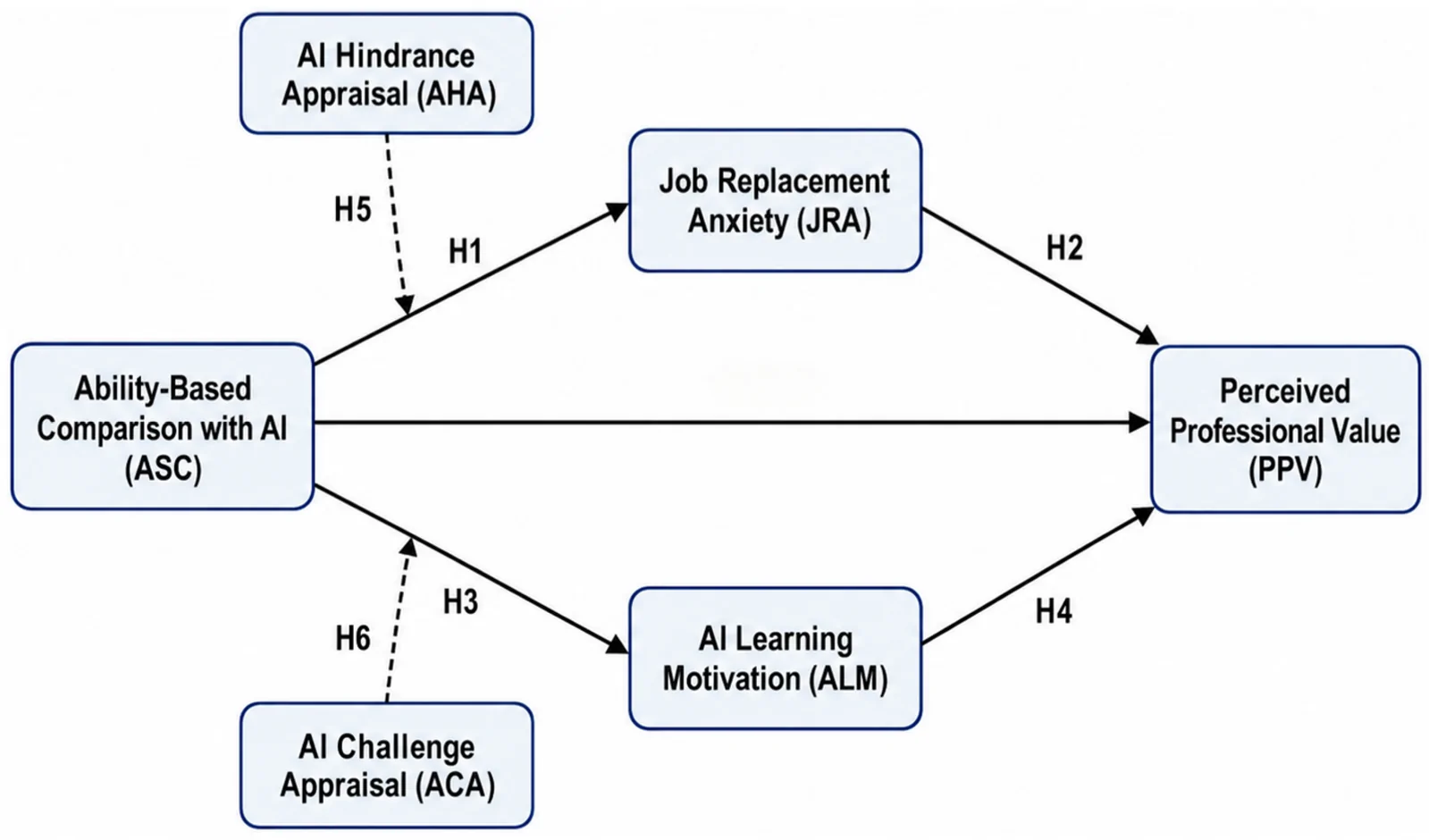
Conceptual model. Note: H1–H6 denote Hypotheses 1–6. Solid arrows indicate direct paths; dashed arrows indicate moderation effects.

The hypothesized relationships are summarized in the conceptual model presented in Figure 1.

## 3. Materials and Methods

### 3.1. Research Design

This study adopted an explanatory sequential mixed-methods design comprising a dominant quantitative phase followed by a supplementary qualitative phase. The quantitative phase used a cross-sectional online questionnaire to examine the relationships among ability-based comparison with generative artificial intelligence, AI appraisal, job replacement anxiety, AI learning motivation, and perceived professional value. Partial least squares structural equation modeling was employed to test the proposed direct, indirect, and moderating relationships. The qualitative phase explored students’ experiences, interpretations, and adaptation strategies regarding generative AI to provide contextual explanations for the quantitative findings. The two forms of evidence were connected and integrated during interpretation, with the qualitative material used to illuminate possible processes, task contexts, and boundary conditions underlying the quantitative patterns rather than to provide participant-level validation of those relationships.

### 3.2. Quantitative Phase

All constructs were operationalized using multi-item scales adapted from established measures and prior empirical research. Item wording was contextually modified to refer to generative AI and journalism and communication education while retaining the intended construct meanings. All responses were measured on a five-point Likert scale. For consistency, the focal constructs are abbreviated as follows: ability-based comparison with AI (ASC), AI challenge appraisal (ACA), AI hindrance appraisal (AHA), job replacement anxiety (JRA), AI learning motivation (ALM), and perceived professional value (PPV).

#### 3.2.1. Participants and Procedure

The target population consisted of university students enrolled in journalism- and communication-related programs in China, including journalism, communication, media studies, advertising, broadcasting and television, network and new media, digital media, and closely related fields. A non-probability sampling strategy combining purposive eligibility screening, convenience recruitment, and participant-assisted snowball distribution was employed. Purposive screening was used to ensure that respondents belonged to the target academic fields, while convenience and snowball recruitment were used to reach eligible students across different institutions and stages of higher education.

The questionnaire was administered through Wenjuanxing (Changsha Ranxing Information Technology Co., Ltd., Changsha, China), an online survey platform widely used in China. The survey link was distributed through academic networks, course groups, and student communities, and participants were invited to forward it to eligible peers. Data were collected between April and May 2026. Before accessing the questionnaire, participants were informed of the purpose of the study, the voluntary nature of participation, the intended use of the data, confidentiality safeguards, and their right to discontinue participation. Participants who read the electronic consent statement and proceeded to submit the questionnaire were considered to have provided informed consent.

A total of 553 responses were initially collected. Data-quality screening was conducted sequentially before analysis using established indicators of careless or insufficient-effort responding, including attention-check failures, unusually short completion times, and highly patterned responses (Meade & Craig, 2012; Niessen et al., 2016). Eight responses were excluded for failing the academic eligibility criteria, 12 for failing either of two embedded attention checks, five for more than 10% missing responses, 11 for completion times shorter than one-third of the sample median, and 10 for highly patterned responding, operationalized in this study as selecting the same response option for at least 80% of the substantive Likert-scale items. Each response was counted only under the first exclusion criterion it met. In total, 46 responses were excluded, yielding a final quantitative sample of 507.

The final sample included 179 men, 321 women, and seven participants who preferred not to disclose their gender. In terms of academic level, the sample comprised 68 first-year undergraduate students, 93 second-year undergraduate students, 113 third-year undergraduate students, 75 fourth-year undergraduate students, 66 first-year master’s students, 56 second-year or above master’s students, 15 first-year doctoral students, 12 second-year doctoral students, and nine third-year or above doctoral students. The institutional composition included 111 students from Project 985 universities, 118 from Project 211 universities excluding Project 985 institutions, 227 from ordinary undergraduate institutions, 24 from vocational or junior colleges, and 27 from other types of institutions.

Participants also differed in their frequency and purposes of generative AI use. Twenty-three respondents reported that they had never used generative AI, 149 reported occasional use of one to three times per week, 182 reported moderate use of four to seven times per week, and 153 reported use more than seven times per week. The most frequently reported application contexts were information searching, writing assistance, coursework support, creative generation, examination preparation, data analysis, and coding or technical learning. These variations provided a heterogeneous quantitative sample in terms of both educational stage and experience with generative AI.

#### 3.2.2. Measures

All focal constructs were assessed using multi-item measures rated on a five-point Likert scale ranging from 1 (strongly disagree) to 5 (strongly agree). The measures were adapted from established theoretical and empirical research and were contextualized for generative AI use in journalism and communication education. The complete wording of all items is provided in Table S1 of the Supplementary Materials.

Ability-based comparison with AI was assessed using three items adapted from research on social comparison orientation and technology-mediated comparison (Gibbons & Buunk, 1999; Vogel et al., 2014). The items captured students’ general tendency to compare their professional performance with generative AI, evaluate whether their abilities were better or worse than AI-generated outputs, and attend to perceived gaps between their own professional abilities and what generative AI could produce. Higher scores indicated a stronger tendency to engage in ability-related comparison with AI. Because the measure focused primarily on the frequency and salience of comparison, rather than its detailed task context or qualitative content, these dimensions were further explored in the interview phase.

AI challenge appraisal and AI hindrance appraisal were measured as two distinct constructs using three items each. The items were adapted and contextually modified primarily from Searle and Auton’s (2015) Challenge and Hindrance Appraisal Scale, with conceptual grounding in the Transactional Model of Stress and Coping and the challenge–hindrance framework (Cavanaugh et al., 2000; Lazarus & Folkman, 1984). The wording was modified to reflect generative AI and students’ professional development in journalism and communication. The AI challenge appraisal assessed the extent to which students viewed generative AI as a manageable demand that could encourage professional development, higher-level capability formation, and constructive adaptation. AI hindrance appraisal assessed the extent to which students viewed generative AI as creating constraints, uncertainty, and obstacles to professional development. The two appraisal dimensions were modeled separately because opportunity-oriented and obstruction-oriented interpretations may coexist rather than constitute opposite ends of a single continuum.

Job replacement anxiety was measured using four items adapted from research on technological replaceability, automation-related concerns, and job insecurity (Brougham & Haar, 2018; Shoss, 2017). The items were contextually modified for students preparing to enter journalism- and communication-related occupations. They assessed anxiety that generative AI might reduce employment opportunities, replace selected professional roles, weaken the continuing need for human professionals, or make entry-level positions less secure. Higher scores indicated stronger anticipatory concern about AI-related occupational displacement.

AI learning motivation was assessed using four items developed with reference to prior research on students’ behavioral intentions to learn AI (Chai et al., 2021, 2023) and conceptually grounded in Self-Determination Theory (Deci & Ryan, 2000; Ryan & Deci, 2020). Prior AI education research has operationalized behavioral intention to learn AI as students’ willingness and self-directed intention to continue developing AI-related knowledge and competencies (Chai et al., 2021, 2023). Consistent with this conceptualization, the four items in the present study assessed students’ willingness, intention, and anticipated effort to acquire generative AI knowledge and skills relevant to journalism and communication work. Specifically, the items addressed motivation to learn how to use generative AI, willingness to invest time in improving one’s ability to work with AI tools, intention to continue learning about relevant AI applications, and willingness to seek training or learning resources. Higher scores indicated stronger motivation to engage in AI-related professional learning.

Perceived professional value was assessed using four items adapted from research on task significance, prosocial impact, and professional identity development (Grant, 2008; Hackman & Oldham, 1976; Trede et al., 2012). The measure captured students’ perceptions of the social importance of journalism and communication, the meaningful social value of their professional knowledge, the contribution of their field to understanding and serving society, and the continuing relevance of their professional training amid technological change. Higher scores indicated stronger perceived value of the professional field and its associated forms of knowledge and competence.

Gender, academic level, institutional background, and frequency of generative AI use were included as control variables because they could plausibly be associated with students’ professional concerns, learning motivation, and perceptions of professional value. Gender and institutional background were dummy-coded, with male respondents and ordinary undergraduate institutions serving as the reference categories. Academic level and generative AI use frequency were treated as ordinal variables. The controls were selected on theoretical grounds rather than through automatic statistical adjustment, consistent with recommendations concerning the justified use of control variables in behavioral research (Atinc et al., 2012; Bernerth & Aguinis, 2016).

#### 3.2.3. Quantitative Data Analysis

Quantitative data analysis was conducted using SmartPLS 4 (SmartPLS GmbH, Monheim am Rhein, Germany) and SPSS Statistics 27 (IBM Corp., Armonk, NY, USA). PLS-SEM was selected because this study aimed not only to test the hypothesized relationships, but also to evaluate the explained variance and predictive relevance of the endogenous constructs while estimating mediation and moderation effects within the same model. Although CB-SEM would also be appropriate for confirmatory theory testing and overall model-fit assessment, PLS-SEM was more closely aligned with the present study’s explanatory and prediction-oriented objectives (Hair et al., 2021).

The analysis was conducted in two stages. First, the measurement model was evaluated to assess the reliability and validity of the constructs. Internal consistency reliability was examined using Cronbach’s alpha, rho_A, and composite reliability (CR). Convergent validity was assessed through standardized factor loadings and average variance extracted (AVE). Discriminant validity was evaluated using the Fornell–Larcker criterion and the heterotrait–monotrait ratio (HTMT).

Second, the structural model was examined to test the hypothesized relationships among ability-based comparison with AI, AI appraisal, job replacement anxiety, AI learning motivation, and perceived professional value. Bootstrapping procedures with 5000 resamples were conducted to estimate the significance of path coefficients and indirect effects. The structural model was evaluated based on standardized path coefficients, explained variance (R^2^), effect sizes (f^2^), predictive relevance (Q^2^), and variance inflation factor (VIF) values.

To further examine the robustness of the mediation and moderation mechanisms, additional analyses were conducted using the PROCESS macro for SPSS (Andrew F. Hayes, Calgary, AB, Canada). Specifically, Model 7 was employed to test the moderated mediation effects, with 5000 bootstrap samples used to generate confidence intervals for conditional indirect effects. Continuous variables were mean-centered before creating interaction terms.

Because the study relied on self-reported cross-sectional data, potential common method variance was assessed before hypothesis testing (P. M. Podsakoff et al., 2003). Harman’s single-factor test was conducted as an initial diagnostic procedure, and full-collinearity variance inflation factor (VIF) values were examined as an additional assessment.

### 3.3. Qualitative Phase

#### 3.3.1. Interview Participants and Procedure

Following the quantitative analysis, the qualitative phase was designed to clarify three findings that required further interpretation: the pronounced anxiety-related pathway, the non-significant moderating effect of AI challenge appraisal on the ASC–ALM relationship, and the remaining positive direct association between ASC and PPV. The interviews also examined the specific tasks, evaluation criteria, and contextual conditions underlying these patterns.

A separate sample of 20 students was recruited for the qualitative phase. Stratified purposive sampling with a maximum-variation orientation was used to ensure variation across academic stages and institutional backgrounds. Although participants were recruited within predefined educational strata, the qualitative sample was not intended to be statistically representative of the quantitative sample. Its purpose was to obtain information-rich accounts from students at different stages of professional education and from different types of higher education institutions.

Eligible participants were required to be at least 18 years old; currently enrolled in journalism, communication, advertising, broadcasting and television, network and new media, digital media, or a related academic program; have prior experience using generative AI; participate voluntarily; and provide explicit consent for audio recording. The final qualitative sample included 10 men and 10 women. It comprised two first-year undergraduate students, two second-year undergraduate students, two third-year undergraduate students, two fourth-year undergraduate students, two first-year master’s students, three second-year or above master’s students, two first-year doctoral students, two second-year doctoral students, and three third-year or above doctoral students.

Semi-structured interviews were conducted in Chinese via Tencent Meeting (Tencent, Shenzhen, China) between 1 August and 6 August 2026, with each interview lasting approximately 30–45 min. All interviews were conducted by the first author, who had no prior relationship with the participants. The interview guide focused on the situations in which students compared themselves with generative AI, the professional tasks and capability dimensions involved, their emotional and cognitive responses to perceived capability differences, and how they interpreted AI as an opportunity, challenge, constraint, or occupational threat. Participants were also asked about concerns regarding job replacement, motivation to acquire AI-related skills, perceptions of changes in the value of journalism and communication education, and professional capabilities they considered difficult to automate. With participants’ consent, the interviews were recorded and automatically transcribed using Tencent Meeting. The automatically generated transcripts were subsequently checked against the audio recordings and manually corrected for transcription errors. Chinese quotations selected for inclusion in the English manuscript were translated by the first author and reviewed by another author for accuracy and consistency with the original meaning. Recruitment ended after 20 interviews. Later interviews primarily repeated patterns identified in earlier interviews and did not introduce new material sufficient to alter the core themes. The resulting dataset was therefore considered thematically adequate for addressing the study’s central research questions.

Interview materials are managed using research codes or pseudonyms. Names and other direct identifiers are removed during transcription, and identifying contextual details are omitted or generalized where necessary. Contact information, oral-consent records, audio recordings, researcher notes, and de-identified transcripts were stored separately and were accessible only to authorized members of the research team. Only de-identified excerpts may be used in manuscripts, presentations, or reports.

#### 3.3.2. Qualitative Data Analysis

The de-identified interview transcripts were imported into NVivo 15 (Lumivero, Denver, CO, USA) for analysis. A codebook-based thematic analysis combining deductive and inductive coding was employed, allowing the qualitative analysis to remain connected to the study’s theoretical model while also identifying experiences and interpretations not anticipated by the survey measures. The initial deductive coding framework was informed by the focal constructs of ability-based comparison with AI, AI challenge appraisal, AI hindrance appraisal, job replacement anxiety, AI learning motivation, and perceived professional value. The inductive component allowed additional codes to emerge from the data, including task-specific comparison contexts, emotional responses, coping strategies, peer-comparison experiences, curricular concerns, and perceptions of distinctively human professional competence.

Two researchers independently coded five transcripts (25% of the qualitative sample). Before reconciliation, intercoder agreement was calculated at the coded-segment level across the preliminary code framework using Cohen’s kappa (κ = 0.84). The researchers then compared coding decisions, discussed discrepancies, clarified code definitions and boundaries, and revised the codebook through consensus. This procedure was used to assess consistency in code application rather than the objective validity of the themes. The first author subsequently coded the remaining 15 transcripts using the revised codebook, documenting ambiguous excerpts for review with the second researcher. Related codes were grouped into candidate themes, which were reviewed for internal coherence, conceptual distinctiveness, and adequate support in the interview material, with attention to similarities and differences across participants and academic levels. Representative quotations were further reviewed to minimize identification risk.

## 4. Results

### 4.1. Descriptive Statistics and Correlations

The mean values of the six focal constructs ranged from 2.952 to 3.662, indicating moderate to moderately high levels across ASC, ACA, AHA, JRA, ALM, and PPV. ALM showed the highest mean score (M = 3.662, SD = 0.982), followed by PPV (M = 3.490, SD = 0.982) and ACA (M = 3.431, SD = 0.987). AHA had the lowest mean score (M = 2.952, SD = 1.038). At the descriptive level, students therefore reported relatively strong motivation to acquire AI-related capabilities while also expressing moderate levels of occupational concern. Skewness and kurtosis values were within acceptable ranges, indicating no serious deviation from normality.

The correlation results provided preliminary support for the proposed competing-pathways framework. ASC was positively correlated with both JRA (r = 0.520, *p* < .01) and ALM (r = 0.504, *p* < .01), indicating that stronger comparison with AI coexisted with both anxiety-related and motivation-related responses. Thus, students who reported greater attention to human–AI capability differences did not display a uniformly negative or positive orientation toward AI.

JRA was negatively correlated with PPV (r = −0.259, *p* < .01), whereas ALM was positively correlated with PPV (r = 0.428, *p* < .01). These opposing correlations were consistent with the expectation that professional value perceptions may be related to both concerns about future occupational displacement and willingness to adapt to technological change. ASC was also positively correlated with PPV (r = 0.256, *p* < .01), suggesting that human–AI comparison may be related to professional value through associations not fully captured by anxiety and learning motivation. This possibility was examined further in the structural model.

ACA and AHA were moderately and positively correlated (r = 0.360, *p* < .01). This pattern indicates that challenge and hindrance appraisals were not mutually exclusive: students could view generative AI as offering opportunities for development while simultaneously perceiving it as a source of uncertainty or obstruction.

### 4.2. Measurement Model

Because all focal constructs were measured using the same self-report questionnaire, common method variance was assessed before testing the structural model. Harman’s single-factor test showed that the first unrotated factor explained 34.43% of the total variance, below the conventional 50% threshold. Full-collinearity diagnostics further showed that VIF values ranged from 1.660 to 2.175, below the recommended threshold of 3.3. These results did not indicate that a single common factor dominated the observed relationships. Nevertheless, because these procedures are diagnostic rather than definitive, the possibility of residual common method variance cannot be completely excluded.

The measurement model was then evaluated for reliability, convergent validity, and discriminant validity. All constructs demonstrated satisfactory internal consistency and convergent validity. Cronbach’s α values ranged from 0.853 to 0.900, rho_A values ranged from 0.855 to 0.900, and composite reliability values ranged from 0.911 to 0.930. Standardized factor loadings ranged from 0.844 to 0.900, and AVE values ranged from 0.750 to 0.783. These values met the recommended criteria, indicating that the items consistently represented their corresponding latent constructs (Hair et al., 2021).

Discriminant validity was further supported by the Fornell–Larcker criterion and HTMT results. The square roots of AVE ranged from 0.866 to 0.885 and exceeded the corresponding interconstruct correlations. HTMT values ranged from 0.276 to 0.641, all below the 0.90 threshold. The HTMT value between ACA and AHA was 0.420, supporting their empirical distinction despite their positive correlation. These findings were consistent with treating challenge and hindrance appraisals as related but distinguishable interpretations of AI-related demands.

Collinearity diagnostics indicated no serious multicollinearity. Latent-variable VIF values ranged from 1.013 to 1.639, and indicator-level VIF values ranged from 2.031 to 2.742. The absence of substantial collinearity indicated that the estimated structural relationships were unlikely to be distorted by excessive overlap among the predictors.

### 4.3. Structural Model

The structural model included gender, academic level, institutional background, and frequency of generative AI use as control variables, with these variables specified as predictors of JRA, ALM, and PPV. In the model including these controls, the core relationships remained statistically significant.

Bootstrapping with 5000 resamples supported the proposed competing-pathway model. ASC was positively associated with JRA (β = 0.416, t = 10.688, *p* < .001), supporting H1. Students who more frequently compared their professional abilities with AI performance reported higher levels of job replacement anxiety. This finding supports the positive association predicted in H1 but does not establish that each student first interpreted capability differences as occupational consequences; that process remains a theoretical interpretation for which the interview data provide contextual evidence.

JRA was negatively associated with PPV (β = −0.593, t = 15.364, *p* < .001), supporting H2. Students reporting higher job replacement anxiety also reported lower perceived professional value. The standardized path coefficient was relatively large within the model, indicating a strong negative association between JRA and PPV after accounting for the other variables included in the model.

ASC was also positively associated with ALM (β = 0.321, t = 8.250, *p* < .001), supporting H3. Students who reported stronger ability comparison with AI also tended to report greater motivation to acquire AI-related knowledge and skills. Comparison with AI was therefore associated not only with perceived threat but also with an orientation toward capability development.

ALM was positively associated with PPV (β = 0.468, t = 12.485, *p* < .001), supporting H4. Students with stronger AI learning motivation tended to evaluate their professional field and training more positively. This pattern is consistent with the view that students may regard AI-related competence as something that can be incorporated into, rather than simply substituted for, their existing professional preparation.

In response to RQ1, the direct association between ASC and PPV was examined after accounting for the anxiety- and motivation-based pathways. The association remained significant and positive (β = 0.330, t = 8.094, *p* < .001). The model therefore did not indicate full mediation. ASC was associated with PPV both directly and indirectly through JRA and ALM.

The remaining direct association should not be interpreted as evidence that comparison with AI necessarily improves students’ professional value perceptions. Rather, it indicates that JRA and ALM did not fully account for the relationship between ASC and PPV. Other processes, such as reflection on the boundaries of AI capability and the distinctive contribution of professional judgment, may also be relevant. These possible interpretations were examined through the qualitative interviews.

The model explained 40.1% of the variance in ALM, 36.6% of the variance in JRA, and 43.8% of the variance in PPV. These values indicate moderate explanatory power while also showing that substantial variation remained attributable to factors outside the proposed model. Q^2^_predict_ values were positive for all endogenous constructs, ranging from 0.210 to 0.393, indicating predictive relevance.

Effect-size results further clarified the relative importance of the predictors. JRA showed the largest association with PPV (f^2^ = 0.451), followed by ALM (f^2^ = 0.287), whereas the direct association between ASC and PPV was smaller (f^2^ = 0.118). This pattern reinforces the importance of the two intervening psychological responses, particularly replacement anxiety, in understanding differences in students’ professional value perceptions.

### 4.4. Mediation Effects

The mediation results further clarified how ASC was related to PPV. Two significant but opposing indirect associations were observed. The indirect association through ALM was positive and significant (β= 0.150, t = 7.354, *p* < .001, 95% CI [0.110, 0.192]), whereas the indirect association through JRA was negative and significant (β = −0.247, t = 8.551, *p* < .001, 95% CI [−0.307, −0.193]). These findings were consistent with the proposed competing-pathways framework.

The positive indirect association indicates that stronger ASC was related to higher ALM, which was in turn related to stronger PPV. In parallel, stronger ASC was related to higher JRA, which was associated with weaker PPV. Human–AI comparison was therefore related to professional value in opposing ways rather than through a single uniformly adaptive or threatening pattern.

At the level of point estimates, the negative indirect association through JRA was larger in absolute magnitude than the positive indirect association through ALM. Together with the larger f^2^ for the association between JRA and PPV, the threat-related pathway was numerically more prominent in the present sample. This comparison is descriptive, however, because the difference between the two indirect effects was not directly tested.

### 4.5. Moderation Effects

The moderation analysis showed an asymmetric pattern. AHA significantly moderated the relationship between ASC and JRA (β = 0.151, t = 3.583, *p* < .001), supporting H5. The positive association between ASC and JRA was stronger among students with higher levels of AHA.

This result indicates that the professional meaning assigned to AI-related capability differences varied across students. When generative AI was more strongly perceived as creating obstacles, uncertainty, or constraints, comparison with AI was more closely associated with replacement anxiety. In contrast, at lower levels of AHA, recognizing a capability difference was less strongly related to occupational concern. The interaction therefore suggests that comparison alone was not equally threatening for all students; its association with JRA depended partly on whether AI was already interpreted as an impediment to professional development.

However, ACA did not significantly moderate the relationship between ASC and ALM (β = −0.029, t = 0.955, *p* = 0.340); therefore, H6 was not supported. Nevertheless, ACA showed a significant positive main association with ALM (β = 0.420, t = 11.664, *p* < .001). Students who interpreted AI-related demands as opportunities for competence development generally reported stronger motivation to acquire AI-related knowledge and skills.

The distinction between the main effect and the interaction effect is important. ACA was positively associated with the overall level of ALM, but no evidence was found that the strength of the association between ASC and ALM varied significantly across levels of ACA. Thus, the significant main association of ACA with ALM should not be interpreted as evidence that challenge appraisal amplified the motivational association between human–AI ability comparison and AI learning motivation.

This pattern suggests that opportunity-oriented appraisal and comparison-based discrepancy recognition may represent partly distinct correlates of AI learning motivation. ACA reflects a general tendency to interpret AI-related change as containing possibilities for mastery and development, whereas ASC may provide diagnostic information about differences between students’ current abilities and emerging professional requirements. Such comparison information may be associated with learning motivation even when students do not strongly frame AI as an opportunity.

The quantitative results alone cannot determine which additional conditions may be associated with the link between perceived capability differences and learning motivation. The qualitative phase therefore examined how participants connected task relevance, external requirements, perceived actionability, and concrete performance feedback with their willingness or efforts to learn AI-related skills, thereby providing possible contextual explanations for the positive association between ASC and ALM and the non-significant interaction between ASC and ACA.

Overall, the quantitative findings were consistent with the proposed competing-pathways model. ASC was associated with PPV through a positive direct association, a positive indirect association through ALM, and a negative indirect association through JRA. The moderation results further showed that the positive association between ASC and JRA was stronger at higher levels of AHA. ACA was positively associated with ALM, but no evidence was found that ACA significantly altered the strength of the association between ASC and ALM.

### 4.6. Qualitative Interpretation and Contextual Integration of the Quantitative Findings

The interview material was used to interpret and contextualize the principal quantitative findings. Initial coding and subsequent code aggregation generated 48 codes, which were further organized into six broader categories concerning AI task integration and efficiency, task-specific ability comparison, risk and responsibility boundaries, occupational and emotional adjustment, adaptive action, and responsibility-oriented human–AI collaboration. For clarity, the findings are reported below through four themes corresponding to the central relationships in the quantitative model. The quotations presented below were drawn from the Chinese interview transcripts and lightly edited for readability without altering their substantive meaning. Quotations included in the English manuscript were translated by the first author and reviewed by another author for accuracy and consistency with the original meaning.

#### 4.6.1. Human–AI Ability Comparison Was Task- and Criterion-Dependent

The interviews clarified that students did not generally compare themselves with generative AI through a global judgment of whether humans or AI were more capable. Comparison became most salient when students and AI worked from similar materials and when the resulting outputs could be evaluated according to relatively explicit criteria, such as speed, structural completeness, linguistic fluency, informational coverage, or the number of alternatives generated.

In standardized production tasks, AI’s advantage was particularly visible. P02 described comparing a manually written report with several AI-generated versions:

“It took me two and a half hours to write the story, whereas AI produced three versions within seconds. The paragraphs were clearly organized, the lead contained all the required elements, and the inverted-pyramid structure was complete. In standardized news writing, I had to admit that it performed better than I did.”

(P02)

Such direct output comparison made AI a concrete and professionally relevant comparison target. However, participants’ evaluations changed when task success depended on contextual knowledge, community-specific language, relational understanding, or information that could not be fully represented in prompts. In these situations, linguistic fluency or structural completeness was not necessarily treated as evidence of superior communication competence.

Across multiple interviews, AI’s advantages in summarization, formatting, initial drafting, translation, structural organization, basic data processing, and the rapid generation of alternatives were repeatedly mentioned. By contrast, human capabilities were more often emphasized when tasks required field access, contextual interpretation, relationship building, knowledge of local audiences, source verification, or responsibility for implementation. These accounts suggest that ASC should not be treated uniformly as an upward comparison; the direction and psychological relevance of comparison varied with the specific professional task and the criteria used to evaluate successful performance.

#### 4.6.2. Task-Level Capability Differences Were Linked to Replacement Anxiety Through Occupational Interpretation

The second theme illustrated how participants connected task-level comparisons with concerns about job replacement. AI was described as capable of efficiently generating initial drafts, summaries, translations, scripts, and analytical frameworks, but such efficiency was not consistently interpreted as an occupational threat. When participants extended the meaning of perceived capability differences from the immediate task to the future value of their own skills, entry-level employment opportunities, and changing recruitment requirements, concerns about replacement tended to be expressed more explicitly.

P19 described this shift:

“Seeing how quickly it writes makes me worry that content operations, basic copywriting, and what résumés often call ‘information integration’ may be devalued. When job advertisements also require proficiency with AI tools and AI-assisted content review, the concern becomes more concrete. I do not think all positions will disappear, but the everyday composition of jobs is being rewritten.”

(P19)

This account linked AI’s efficiency in routine production to the changing occupational value of entry-level skills. Participants were concerned less with the complete disappearance of journalism and communication work than with the contraction or reorganization of standardized content-production and information-processing tasks. They anticipated that fewer workers might be needed for some routine activities, while entry-level positions would increasingly require broader combinations of content production, AI operation, data literacy, platform knowledge, audience analysis, and project coordination.

The association between comparison and replacement anxiety appeared more pronounced when participants perceived limited access to relevant training, uncertainty about which capabilities would remain valuable, or little control over occupational change. Under these conditions, AI’s performance was more readily interpreted as evidence that existing skills and educational investment were losing value. Hindrance appraisal therefore appeared to shape the occupational meaning assigned to the comparison gap.

Participants also described an important boundary condition. Some distinguished the contraction of standardized entry-level tasks from the disappearance of the profession as a whole, particularly when work involved field research, customized planning, source or client communication, and context-dependent judgment. For these students, AI’s advantage was associated with concern about particular tasks or career entry points without producing a generalized judgment that journalism and communication had become professionally redundant.

The qualitative findings therefore suggest a possible interpretive sequence underlying the ASC–JRA association. Comparison was more closely associated with replacement anxiety when AI’s task-level advantage was interpreted as evidence of declining occupational demand and when students perceived limited control over adaptation. When AI’s advantage was confined to routine tasks and students could identify other professionally consequential capabilities, the same comparison was associated with less generalized anxiety.

#### 4.6.3. Comparison Was Linked to Learning Motivation Through Diagnosed Gaps and Concrete Task Demands

The third theme offered a possible explanation for why ASC was positively associated with AI learning motivation and why ACA did not significantly strengthen this association. Participants often described comparison as a diagnostic process. AI-generated outputs made specific gaps in structural organization, information processing, prompt design, data handling, verification procedures, or workflow efficiency more visible. However, recognizing a capability gap was not necessarily accompanied by sustained learning motivation or learning action.

In many accounts, movement from recognition to action was connected with course requirements, research tasks, internship deadlines, team responsibilities, or workflow problems that required an immediate response. P17 explained:

“Without an external schedule, I could easily put learning AI off until ‘later.’ One course required us to analyze the same communication material using different methods and explain where AI was used, what prompts were given, how the results were checked, and what the limitations were. That requirement turned vague interest into a clear schedule and a concrete deliverable.”

(P17)

This assignment made AI-related learning more actionable by providing a timeline, assessable deliverables, and explicit requirements for documenting and evaluating AI use. In this account, practical relevance appeared to be more closely connected with movement from general interest toward a more systematic and responsible workflow than positive appraisal alone.

Two distinguishable patterns of learning narratives appeared across the interview material. The first was opportunity-oriented. In these accounts, AI performance was interpreted as representing a standard that could be approached through learning, thereby making possibilities for mastery and professional development more salient. Participants who described AI in opportunity-oriented terms often also expressed stronger interest and willingness to learn, although positive appraisal did not necessarily correspond to sustained learning action.

The second was a necessity-oriented route, in which comparison exposed a capability gap that interfered with an immediate task or anticipated professional requirement. Participants could remain concerned about reliability, privacy, dependence, or occupational displacement while still recognizing that the ability to use and evaluate AI had become necessary for coursework, research, internships, or recruitment. In these cases, learning was associated less with an attractive challenge frame than with the practical consequences of leaving a relevant capability gap unresolved.

These accounts help interpret the non-significant ASC × ACA interaction. ACA appeared to provide a generally favorable orientation toward AI learning, whereas deadlines, assessable outputs, recurring workflow problems, and professional relevance appeared more important in making a particular discrepancy actionable. Because necessity-oriented adaptation did not require strong challenge appraisal, the association between ASC and ALM could remain present across different levels of ACA. This interpretation is consistent with the additive rather than interactive pattern observed in the quantitative analysis.

#### 4.6.4. Comparison with AI Was Associated with a More Specific Understanding of Professional Value

The fourth theme offered a possible interpretation of why ASC retained a positive direct association with PPV after JRA and ALM were included in the quantitative model. Participants’ accounts suggested that comparison with AI was associated not only with anxiety and the identification of new technological skills, but also with reconsideration of what journalism and communication competence consisted of.

When AI performed well in writing, summarization, translation, information organization, and structural production, students sometimes became less confident in the distinctiveness of routine production abilities. At the same time, comparison made other forms of professional work more visible, including defining communication problems, evaluating evidence, understanding why people did or did not speak, identifying affected groups, interpreting context, assessing potential harm, and assuming responsibility for communication outcomes.

P09 described this shift in the context of health communication:

“AI made me more clearly understand that professional value lies in judging the public consequences of information: who will see it, who may be excluded, whether individual responsibility is being overstated, and whether differences in access to services are being concealed. Source verification, ethics, and empathy are not decorative elements in health communication.”

(P09)

P09’s account suggests that AI’s productive efficiency was not interpreted simply as evidence of declining professional value. Rather, it was accompanied by recognition of the limitations of defining journalism and communication primarily in terms of efficient content production. Across other accounts, participants similarly associated the continuing value of the field with evidence evaluation, contextual understanding, audience differences, ethical judgment, interpersonal relationships, and responsibility for final decisions.

P15 made this distinction particularly explicit:

“The faster AI generates content, the more I care about whom it cites, whom it leaves out, and what kind of common sense it uses to speak for others. My understanding of professional value did not become weaker; it became more concrete. Journalism judgment, source verification, contextual understanding, ethics, public responsibility, and interpersonal communication still matter, but they have to be practised rather than treated as automatic human advantages.”

(P15)

This account also illustrates an important qualification. Participants did not simply construct an idealized division in which AI performed technical work while humans naturally possessed ethics, empathy, and judgment. Several accounts acknowledged that human professionals could also reproduce stereotypes, overlook less visible groups, misinterpret context, or avoid responsibility. These capabilities were therefore discussed not as automatic human advantages, but as professional competencies requiring education, institutional norms, sustained practice, and accountability.

The positive direct association between ASC and PPV is therefore consistent with participants’ accounts in which professional value was defined in more specific terms. In some interviews, the perceived exclusivity of routine production skills was described as declining, while the limitations of speed, fluency, and formal completeness as criteria of professional competence were more explicitly recognized. Participants instead linked the continuing social and occupational value of journalism and communication to evidence evaluation, contextual understanding, audience judgment, ethical responsibility, interpersonal engagement, and accountability for final decisions.

Taken together, the four themes contextualize the competing-pathways model by showing that the meaning assigned to human–AI comparison varied with task criteria, occupational interpretation, the perceived actionability of capability differences, and understandings of professional responsibility. The interview material does not establish these patterns as causal mechanisms underlying the quantitative relationships, but provides contextual explanations for how threatening, adaptive, and professional-value-related interpretations could coexist across participants’ accounts.

## 5. Discussion

### 5.1. Principal Findings and Mixed-Methods Interpretation

This study examined how ability-based comparison with generative AI was associated with journalism and communication students’ perceived professional value. The quantitative results revealed three simultaneous patterns: a negative indirect association through job replacement anxiety, a positive indirect association through AI learning motivation, and a positive direct association between ability-based comparison and perceived professional value. The interviews offered contextual interpretations of why the same comparison tendency could be related to these different outcomes by identifying variation in the tasks being compared, the occupational meaning assigned to capability differences, the practical conditions of learning, and the boundaries students drew around professional responsibility.

First, human–AI comparison was task-contingent and multidimensional. The survey measure of ability-based comparison captured students’ tendency to attend to and evaluate differences between their own capabilities and AI performance; it did not assume that AI was superior across all professional domains. The interviews showed that AI was frequently perceived as stronger in speed, information compression, structural organization, standardized drafting, and the production of multiple alternatives. However, students often evaluated human capability more positively when tasks required field access, contextual knowledge, interpersonal communication, source verification, interpretation of ambiguity, or responsibility for consequences. AI therefore became a meaningful comparison target only in relation to particular tasks and evaluation criteria.

This task contingency helps interpret why comparison with AI was related to both anxiety and learning motivation. When students focused on speed or standardized output, AI performance made perceived capability gaps highly visible. When they evaluated the complete professional process, the comparison became more differentiated. An AI-generated text could be formally coherent while failing to represent local knowledge, conflicting evidence, audience differences, or the practical constraints of implementation. The psychological meaning of comparison therefore did not reside in AI performance alone; it depended on which aspect of professional competence students treated as relevant and diagnostic.

Second, the interviews offered a possible interpretation of why the anxiety-related pathway was particularly pronounced. The negative indirect association through JRA was numerically larger than the positive indirect association through ALM. Participants’ accounts indicated that task-level capability differences were more likely to be interpreted as occupationally threatening when students connected them to the possible contraction of entry-level work, the declining value of routine production skills, or increasingly complex recruitment requirements.

This process involved more than observing that AI could complete a task efficiently. Students extended the comparison from the immediate output to the occupational return on their training. Writing, transcription, summarization, information organization, and standardized content adaptation were not understood merely as classroom exercises; they were also associated with internship responsibilities and common entry points into journalism- and communication-related employment. When AI appeared capable of completing these tasks rapidly, some students questioned whether the abilities they were developing would continue to provide access to secure and meaningful work. JRA was therefore closely related to PPV because occupational uncertainty also raised questions about whether professional education remained worth sustained investment.

Chinese higher education and employment policies provided a concrete institutional context for this occupational interpretation. National policy is accelerating the integration of AI into university curricula, discipline-specific teaching, and employment-related capability development, while employment policy increasingly emphasizes closer feedback between industry demand, university training, and graduate career services (General Office of the Communist Party of China Central Committee & General Office of the State Council, 2024; Ministry of Education of the People’s Republic of China et al., 2025). Within this institutional environment, participants frequently interpreted AI learning not as an optional extracurricular interest but as part of changing expectations regarding professional preparation.

At the same time, the interviews identified an important boundary condition. Students did not necessarily generalize AI’s advantage in routine production into the conclusion that journalism and communication had lost all professional value. Some anticipated that standardized entry-level tasks might contract but continued to distinguish these activities from field research, client or source communication, contextual interpretation, audience analysis, and responsibility-bearing decisions. Where students could restrict AI’s advantage to a specific part of the workflow, comparison was associated with concern about particular skills without producing a global judgment that the profession had become obsolete.

Third, the significant moderation by AHA clarifies how occupational interpretation shaped the anxiety-related pathway. The positive association between ASC and JRA remained present independently of the interaction term; AHA was therefore not a necessary condition for comparison to be associated with replacement anxiety. Rather, higher AHA strengthened this association by making capability differences more likely to be interpreted as obstacles to professional development.

The interview accounts suggested that this amplification occurred when students perceived limited control over technological change, uncertainty about which capabilities remained valuable, insufficient time or resources for adaptation, or a mismatch between curricular training and occupational requirements. In these situations, the comparison gap was more readily extended from “AI performs this task effectively” to “my existing skills and future opportunities are becoming less secure.” Hindrance appraisal therefore changed the professional meaning attached to the comparison rather than simply adding another source of anxiety.

Fourth, the motivation-related pathway involved both opportunity-oriented and necessity-oriented adaptation. ASC made discrepancies in writing structure, information processing, data handling, prompt design, verification, and workflow efficiency more visible. However, the interviews showed that recognizing such a discrepancy did not require students to interpret AI positively before it could become motivational. Courses, assessment requirements, research projects, internship responsibilities, and employment expectations could also convert a perceived capability gap into an immediate learning requirement.

This distinction helps explain why ACA had a positive main association with ALM but did not significantly moderate the ASC–ALM relationship. H6 assumed that challenge appraisal would strengthen the extent to which comparison information was translated into motivation. The findings instead distinguish a level effect from an amplification effect. Students with stronger ACA generally reported higher ALM, indicating that an opportunity-oriented interpretation of AI supported learning readiness. However, ACA did not make students substantially more responsive to capability discrepancies revealed through comparison.

This result offers a qualified interpretation of how the Transactional Model of Stress and Coping may apply to AI-related learning contexts, one that requires further empirical testing. Challenge appraisal captures whether an AI-related demand is interpreted as containing possibilities for mastery or gain, but the transition from appraisal to motivated action may also require secondary judgments about controllability, available resources, attainability, and the instrumental value of learning. A student may recognize AI as an opportunity without possessing a concrete reason, schedule, or pathway for sustained learning. Conversely, a student who remains ambivalent or concerned about AI may nevertheless learn when a capability gap interferes with a valued task or creates foreseeable academic and occupational costs.

The non-significant interaction therefore suggests that positive appraisal is one route to AI learning motivation rather than a necessary condition for comparison-based adaptation. In the present sample, ACA provided a generally favorable orientation toward learning, whereas ASC could activate motivation through the diagnostic and practical significance of a capability gap. Adaptive responses to AI may consequently be understood as adjustments to changing professional demands (Sternberg, 2019), emerging through both opportunity-based engagement and necessity-based adjustment.

Finally, the positive direct association between ASC and PPV indicates that JRA and ALM did not fully account for the relationship between the two constructs. The interview material suggests that a more specific articulation of professional value may provide one possible interpretation of this remaining association. In the interview accounts, AI’s advantages in speed, linguistic fluency, and routine content generation were associated with reduced perceived distinctiveness of some conventional production skills, while other dimensions of professional competence were emphasized more explicitly, including defining communication problems, verifying evidence, interpreting context, recognizing excluded perspectives, assessing potential harm, and accepting responsibility for final decisions.

This interpretation should not be understood as professional identity reconstruction because the study did not measure students’ identification with a professional role. PPV concerns whether journalism and communication knowledge, training, and prospective work continue to possess social and occupational value. The interview material showed that some participants articulated professional value more specifically when discussing comparisons with AI. Their accounts did not assume that every human capability was inherently irreplaceable; instead, they placed greater emphasis on evidence evaluation, contextual understanding, relationships, audience differences, ethical judgment, and public accountability.

Taken together, the mixed-methods findings suggest that human–AI comparison did not carry a fixed positive or negative meaning. Task criteria shaped whether AI performance was treated as a meaningful capability difference; occupational interpretation shaped whether that difference was associated with replacement anxiety; concrete demands shaped whether it became a learning target; and reflection on evidence and responsibility shaped whether students questioned or reconstructed perceived professional value.

### 5.2. Theoretical Contributions

The first contribution lies in placing threat-oriented and adaptation-oriented responses within the same framework of ability self-evaluation. Rather than simply documenting positive and negative associations with AI, the findings situate both forms of response within a common comparison framework. The interview material further suggests that occupational interpretations, perceived actionability, and concrete task demands may contribute to how human–AI ability comparisons acquire different psychological meanings.

The anxiety- and motivation-related associations should therefore not be understood as representing two mutually exclusive groups of students. Theoretically, the same student may report both job replacement anxiety and AI learning motivation; these constructs were modeled as distinct responses rather than as opposite ends of a single continuum, and the interview material also contained accounts in which concern about occupational displacement coexisted with motivation to acquire AI-related capabilities. Similarly, ACA and AHA represent distinguishable but non-mutually exclusive forms of appraisal, allowing opportunities for development and perceived obstacles to professional adaptation to coexist.

This interpretation extends the distinction between comparison-based threats and self-improvement. Upward comparison can undermine self-evaluation when an observed difference appears threatening or unattainable, but it can also identify possible directions for development (Lockwood & Kunda, 1997). The present findings suggest that an adaptive orientation toward AI need not depend exclusively on positively assimilating the comparison target. The interview material indicates that external requirements and perceived professional necessity may connect perceived capability gaps with more explicit learning intentions, concrete learning plans, or, in some accounts, reported learning actions.

The second contribution concerns the asymmetric functions of challenge and hindrance appraisal. Hindrance appraisal operated as a conditional amplifier: it strengthened the association between human–AI comparison and replacement anxiety by making capability differences more likely to be interpreted as uncontrollable obstacles to professional development. Challenge appraisal, however, operated primarily as an independent positive orientation. It was associated with higher learning motivation but did not significantly increase the motivational effect of comparison.

This asymmetry indicates that challenge and hindrance appraisals should not be treated as mirror-image boundary conditions. The movement from a task-level capability difference to occupational anxiety depended strongly on whether the difference was interpreted as obstruction and anticipated loss. The movement from a capability difference to learning motivation was less dependent on positive appraisal because adaptation could also be activated by task requirements, performance feedback, and professional necessity.

The findings therefore refine the assumption that challenge appraisal automatically converts demanding information into motivated action. Appraising AI as an opportunity may increase general learning readiness, but comparison-based learning also requires the discrepancy to be relevant, actionable, and instrumentally connected to valued outcomes. In this sense, AI-related adaptation can occur through both positively framed mastery and necessity-driven capability adjustment.

The third theoretical contribution is a task-contingent account of generative AI as a non-human comparison target. Social Comparison Theory has primarily examined self-evaluation in relation to human referents, including peers, role models, and socially visible performers (Buunk & Gibbons, 2007; Festinger, 1954). The present study shows that AI-generated performance can also become self-relevant ability information. However, the contribution is not that AI constitutes a reliable or objective benchmark. The findings instead demonstrate that its relevance as a comparison target depends on the task, the information supplied, the evaluation criterion, and students’ capacity to inspect and revise its output.

Generative AI also differs from a fixed human comparison target because its performance is interactive and partially manipulable. Students can alter prompts, add source materials, select another system, expose errors, and redefine what counts as successful performance. The comparison target is therefore co-produced through human–AI interaction. This makes human–AI comparison simultaneously more immediate and less stable than conventional interpersonal comparisons.

### 5.3. Implications for Journalism and Communication Education

The findings suggest that AI education should be organized around differentiated professional tasks rather than generic tool proficiency. Students need structured opportunities to compare human and AI performance in drafting, source verification, interviewing, audience analysis, data organization, ethical decision-making, and publication. The goal is not to establish the overall superiority of either humans or AI, but to clarify which tasks can be assisted, which outputs require verification, which decisions should remain human-led, and who is accountable for the final result.

As Chinese higher education policy promotes the integration of AI into curricula and discipline-specific training (Ministry of Education of the People’s Republic of China et al., 2025), institutional efforts should extend beyond providing access to tools (Jin et al., 2025; McDonald et al., 2025). Students should learn to document source materials, prompts, evaluation criteria, verification procedures, and identified limitations. AI learning should also be embedded in authentic and accountable workflows (D. Y. Long et al., 2026; Suh, 2025). The non-significant interaction between ACA and ASC indicates that this study did not find evidence that challenge appraisal significantly strengthened the association between ability-based comparison and AI learning motivation. This finding does not negate the positive main association between ACA and ALM, nor does it demonstrate that positive appraisal is ineffective for sustained learning. The interview material further suggests that assignments, deadlines, feedback, and professional relevance may provide concrete conditions linking awareness of capability gaps with learning intentions, learning plans, or reported learning actions. Accordingly, course design could incorporate tasks that require students to identify AI errors, revise human–AI workflows, document verification procedures, and justify both final outputs and the decision processes through which those outputs were produced.

Relevant activities may include comparing human and AI analyses, checking generated summaries against original materials, identifying unsupported references, documenting prompt and revision histories, and assessing effects on different audiences. In this context, AI literacy includes not only tool operation but also information evaluation, uncertainty recognition, data protection, and claim verification (Chan, 2023; Kasneci et al., 2023; W. Yan et al., 2025).

Curriculum reform should further be connected to career guidance and occupational uncertainty. Participants related replacement concerns to changing entry-level tasks, expanding recruitment requirements, and doubts about the occupational value of conventional training. As Chinese employment policy increasingly emphasizes aligning university education and career services with industry demand (General Office of the Communist Party of China Central Committee & General Office of the State Council, 2024), journalism and communication programs should explain more clearly how AI is reshaping professional roles and skill combinations. Neither replacement-only nor opportunity-only narratives are sufficient. Programs should provide realistic information while continuing to strengthen field engagement, interviewing, source verification, contextual interpretation, audience understanding, data and algorithmic literacy, ethical reasoning, and public accountability. These capabilities should not be treated as automatic human advantages, but as competencies requiring sustained training, institutional standards, and responsibility for communication outcomes.

### 5.4. Limitations and Future Research

Several limitations define the scope of the findings. First, the quantitative phase used a cross-sectional, simultaneous self-report design. The observed paths therefore represent associations rather than established temporal or causal processes. Statistical common-method diagnostics cannot eliminate the effects of response style, general AI attitudes, or temporary affect. Longitudinal, experimental, and behavioral designs could examine how comparison experiences develop into anxiety, learning, and professional-value judgments over time.

Second, convenience sampling, snowball distribution, and voluntary participation may have overrepresented students who were more digitally active or interested in AI. The sample was limited to journalism and communication students in China and should not be treated as representative of all students or professional populations. In addition, 23 respondents reported no direct use of generative AI. They may have encountered AI outputs indirectly, but indirect exposure was not measured, leaving uncertainty about the informational basis of their judgments.

Third, the measures have conceptual and validation boundaries. All scales were adapted for the generative AI and journalism and communication education context. Although the measurement model demonstrated satisfactory reliability, convergent validity, and discriminant validity in the present sample, the adapted wording was not subjected to formal expert review, cognitive interviewing, or independent pilot testing before administration. The content validity and generalizability of these measures therefore require further examination in independent samples and across other disciplinary and cultural contexts.

Finally, the interview participants were recruited separately from the survey respondents. The interview accounts provided contextual and process-oriented interpretations of the quantitative patterns, but they cannot establish that the same processes occurred among individual survey respondents or indicate how prevalent the identified patterns were in the quantitative sample. In addition, each interview lasted approximately 30–45 min, and the 20 participants were distributed across multiple academic stages. The qualitative material was therefore better suited to supporting thematic adequacy around the core research questions than to providing equally detailed explanations for each educational stage. Future longitudinal research could link quantitative profiles with subsequent interviews and task-based evidence to examine how comparison, appraisal, anxiety, learning motivation, and professional-value evaluations develop over time.

## 6. Conclusions

This study examined how ability-based comparison with generative artificial intelligence was associated with perceived professional value among journalism and communication students in China. Ability-based comparison with generative artificial intelligence was associated with lower perceived professional value indirectly through higher job replacement anxiety and with higher perceived professional value indirectly through greater artificial intelligence learning motivation, while a positive direct association between ability-based comparison with generative artificial intelligence and perceived professional value remained. The positive association between ability-based comparison with generative artificial intelligence and job replacement anxiety was stronger at higher levels of artificial intelligence hindrance appraisal. Artificial intelligence challenge appraisal was positively associated with artificial intelligence learning motivation, but it did not significantly alter the strength of the association between ability-based comparison with generative artificial intelligence and artificial intelligence learning motivation.

The interview material suggests that the meaning of these quantitative patterns varied with the specific task and with how participants interpreted perceived capability differences. When participants connected artificial intelligence advantages with occupational devaluation and reduced access to entry-level opportunities, they were more likely to describe those differences in threatening terms. When explicit academic or professional requirements made adaptation necessary, capability differences were more likely to be understood as learning targets that required a response. Some participants also connected the human–artificial intelligence comparison with a distinction between routine production capabilities and professional capabilities involving evidence evaluation, contextual understanding, audience judgment, ethical reasoning, and responsibility for communication outcomes.

For journalism and communication education, the central issue is therefore not to determine in the abstract whether students or artificial intelligence performs better, but to help students interpret capability differences within specific professional tasks without equating efficiency with professional value. Educational practice should support the development of both the technical capabilities required for responsible artificial intelligence-assisted work and the professional capacities needed to evaluate evidence, understand context and audiences, exercise ethical judgment, and remain accountable for communication decisions.

## Data Availability

The de-identified quantitative dataset supporting the findings of this study is available from the corresponding author upon reasonable request. Full interview transcripts are not publicly available because, despite anonymization, the detailed academic and professional experiences described by participants may create a risk of deductive identification. The qualitative coding framework and selected de-identified excerpts may be made available from the corresponding author upon reasonable request, subject to institutional ethical requirements and the protection of participant confidentiality.

## Supplementary Materials

The following supporting information can be downloaded at: https://www.mdpi.com/article/10.3390/jintelligence14090201/s1, Table S1: Measurement items and constructs; Table S2: Demographic characteristics of respondents; Table S3: Descriptive statistics of key constructs; Table S4: Correlations among key constructs; Table S5: Reliability and convergent validity; Table S6: Fornell–Larcker criterion; Table S7: HTMT values; Table S8: Collinearity diagnostics; Table S9: Explanatory power and predictive relevance; Table S10: Effect sizes; Table S11: Direct effects; Table S12: Mediation effects; Table S13: Moderation effects; Table S14: PROCESS robustness check: anxiety-based pathway; Table S15: Conditional indirect effects: anxiety-based pathway; Table S16: Index of moderated mediation: anxiety-based pathway; Table S17: Summary of hypothesis testing; Table S18: PROCESS robustness check: motivation-based pathway; Table S19: Robustness check using PROCESS Model 7; Table S20: Conditional indirect effects: motivation-based pathway; Table S21: Index of moderated mediation: motivation-based pathway; Table S22: Common method bias diagnostics; Table S23: Full collinearity VIF values; Table S24: Effects of control variables on endogenous constructs; Table S25. Characteristics of Interview Participants; Table S26. Qualitative Coding Framework and Theme Development; Figure S1: Structural model with standardized path coefficients.
