# Supplementary material for: Comparing Human Ability with Generative AI: Self-Evaluation, Appraisal, and Adaptive Professional Intelligence Among Journalism and Communication Students
Source: J Intell. 2026 Sep 1;14(9):201. doi: 10.3390/jintelligence14090201 (PMC13608180; doi:10.3390/jintelligence14090201)
Supplement: Supplementary file 1 [file jintelligence-14-00201-s001.zip › jintelligence-4386854-supplementary.pdf]

Supplementary Materials

Supplementary Note:

This supplementary file reports additional statistical results for the study. The analyses include descriptive statistics, correlations, measurement model assessment, structural model assessment, mediation and moderation effects, PROCESS robustness checks, common method bias diagnostics, and control-variable analyses.

All focal constructs were measured on five-point Likert scales. ASC = Ability-Based Comparison with AI; ACA = AI Challenge Appraisal; AHA = AI Hindrance Appraisal; JRA = Job Replacement Anxiety; ALM = AI Learning Motivation; PPV = Perceived Professional Value.

Table S1. Measurement Items and Constructs

Construct	Code	Measurement Items
Ability-Based Comparison with AI (ASC)	ASC1	I often compare my performance with AI in professional tasks
	ASC2	When using generative AI, I tend to evaluate whether my abilities are better or worse than its output
	ASC3	I pay attention to the gap between my own professional abilities and what generative AI can produce
AI Challenge Appraisal (ACA)	ACA1	I see generative AI as a challenge that can push me to develop higher-level professional abilities.
	ACA2	The development of generative AI makes me think more actively about how to improve my professional strengths.
	ACA3	Generative AI creates new demands that I believe I can respond to through professional development.
AI Hindrance Appraisal (AHA)	AHA1	I see generative AI as creating constraints that make professional development in my field more difficult.
	AHA2	The rapid development of generative AI makes it

Construct	Code	Measurement Items
		harder for me to judge which professional abilities are worth developing.
	AHA3	Generative AI makes the standards for professional competence in journalism and communication feel more uncertain.
Job Replacement Anxiety (JRA)	JRA1	I feel anxious that AI may reduce the number of job opportunities available in journalism and communication.
	JRA2	I worry that some professional roles in my field may be replaced by AI systems.
	JRA3	I feel uneasy about whether human professionals will remain needed in some areas of journalism and communication.
	JRA4	I am concerned that AI may make entry-level positions in my field less secure.
AI Learning Motivation (ALM)	ALM1	I am motivated to learn how to use generative AI in journalism and communication work.
	ALM2	I am willing to spend time improving my ability to work with generative AI tools
	ALM3	I intend to keep learning about AI applications relevant to my field.
	ALM4	I would actively seek training or resources that help me use AI more effectively in professional tasks.
Perceived Professional Value (PPV)	PPV1	I believe journalism and communication remain socially important fields.
	PPV2	The professional knowledge I am developing in my major has meaningful social value.
	PPV3	My major helps cultivate abilities that are important for understanding and serving society.
	PPV4	I am confident that the professional training in my field will remain valuable amid technological change.

Table S2. Demographic Characteristics of Respondents

Characteristic	Category	n	%
Gender	Male	179	35.31
	Female	321	63.31
	Prefer not to disclose	7	1.38
Academic level	First-year undergraduate	68	13.41
	Second-year undergraduate	93	18.34
	Third-year undergraduate	113	22.29
	Fourth-year undergraduate	75	14.79
	First-year master’s student	66	13.02

Characteristic	Category	n	%
	Second-year or above master's student	56	11.05
	First-year doctoral student	15	2.96
	Second-year doctoral student	12	2.37
	Third-year or above doctoral student	9	1.78
Institutional background	Project 985 university	111	21.89
	Project 211 university, excluding Project 985	118	23.27
	Ordinary undergraduate institution	227	44.77
	Vocational or junior college	24	4.73
	Other	27	5.33
Generative AI use frequency	Never used	23	4.54
	Occasional use, 1–3 times per week	149	29.39
	Moderate use, 4–7 times per week	182	35.90
	Frequent use, more than 7 times per week	153	30.18
Main AI-use scenario	Information searching	307	60.55
	Writing assistance	286	56.41
	Coursework support	278	54.83
	Creative generation	222	43.79
	Exam preparation	145	28.60
	Data analysis	122	24.06
	Coding or technical learning	84	16.57
	Other	47	9.27

Note. N = 507. Percentages for main AI-use scenarios exceed 100% because respondents could select multiple scenarios.

Table S3. Descriptive Statistics of Key Constructs

Construct	N	Min	Max	Mean	SD	Skewness	Kurtosis
ASC	507	1	5	3.036	1.020	0.010	-0.707
ACA	507	1	5	3.431	0.987	-0.255	-0.657
AHA	507	1	5	2.952	1.038	0.101	-0.736
JRA	507	1	5	3.173	0.996	0.047	-0.671
ALM	507	1	5	3.662	0.982	-0.419	-0.625
PPV	507	1	5	3.490	0.982	-0.263	-0.707

Table S4. Correlations among Key Constructs

Construct	1	2	3	4	5	6
1. ASC	1					
2. ACA	.433**	1				
3. AHA	.399**	.360**	1			
4. JRA	.520**	.327**	.460**	1		

Construct	1	2	3	4	5	6
5. ALM	.504**	.561**	.310**	.348**	1	
6. PPV	.256**	.290**	-.241**	-.259**	.428**	1

Note. **p < .01.

Table S5. Reliability and Convergent Validity

Construct	Items	Loading Range	Cronbach's α	CR (rho_A)	CR (rho_C)	AVE
ACA	ACA1–ACA3	.871–.891	.853	.855	.911	.773
AHA	AHA1–AHA3	.875–.900	.861	.861	.915	.783
ALM	ALM1–ALM4	.869–.882	.900	.900	.930	.769
ASC	ASC1–ASC3	.880–.888	.861	.862	.915	.782
JRA	JRA1–JRA4	.844–.891	.896	.898	.928	.763
PPV	PPV1–PPV4	.857–.879	.889	.889	.923	.750

Table S6. Fornell–Larcker Criterion

Construct	ACA	AHA	ALM	ASC	JRA	PPV
ACA	.879					
AHA	.360	.885				
ALM	.562	.310	.877			
ASC	.433	.400	.504	.884		
JRA	.326	.461	.348	.521	.874	
PPV	.292	-.241	.428	.257	-.259	.866

Note. Diagonal values are the square roots of AVE.

Table S7. HTMT Values

Construct Pair	HTMT
AHA–ACA	.420
ALM–ACA	.641
ALM–AHA	.352
ASC–ACA	.505
ASC–AHA	.464
ASC–ALM	.571
JRA–ACA	.373
JRA–AHA	.524
JRA–ALM	.387
JRA–ASC	.591
PPV–ACA	.334
PPV–AHA	.276

Construct Pair	HTMT
PPV–ALM	.478
PPV–ASC	.293
PPV–JRA	.290

Table S8. Collinearity Diagnostics

Diagnostic	Range	Criterion	Interpretation
Latent-variable VIF	1.013–1.639	< 5	No serious multicollinearity
Indicator-level VIF	2.031–2.742	< 5	No serious multicollinearity

Table S9. Explanatory Power and Predictive Relevance

Endogenous Construct	R^2	Interpretation	Q^2predict
ALM	.401	Moderate	.393
JRA	.366	Moderate	.353
PPV	.438	Moderate	.210

Table S10. Effect Sizes

Predictor → Outcome	f^2
ACA → ALM	.236
AHA → JRA	.120
ASC → ALM	.140
ASC → JRA	.227
ASC → PPV	.118
ALM → PPV	.287
JRA → PPV	.451
ACA × ASC → ALM	.001
AHA × ASC → JRA	.029

Table S11. Direct Effects

Path	β	t	p	95% CI
ASC → JRA	.416	10.688	< .001	[.340, .491]
JRA → PPV	-.593	15.364	< .001	[-.668, -.520]
ASC → ALM	.321	8.250	< .001	[.243, .397]
ALM → PPV	.468	12.485	< .001	[.394, .539]
ASC → PPV	.330	8.094	< .001	[.250, .410]
ACA → ALM	.420	11.664	< .001	[.349, .490]
AHA → JRA	.301	7.690	< .001	[.225, .377]

Table S12. Mediation Effects

Indirect Path	Effect	t	p	95% CI
ASC → ALM → PPV	.150	7.354	< .001	[.110, .192]
ASC → JRA → PPV	-.247	8.551	< .001	[-.307, -.193]
ACA → ALM → PPV	.196	7.638	< .001	[.150, .250]
AHA → JRA → PPV	-.178	6.091	< .001	[-.239, -.125]

Table S13. Moderation Effects

Moderating Path	Interaction β	t	p	95% CI	Interpretation
ACA × ASC → ALM	-.029	.955	.340	[-.086, .031]	Not significant
AHA × ASC → JRA	.151	3.583	< .001	[.068, .233]	Significant

Table S14. PROCESS Robustness Check: Anxiety-Based Pathway
Model specification: ASC → JRA → PPV, with AHA moderating the first-stage path.

Predictor	Outcome: JRA	B	t
Constant	JRA	3.114	80.469**
ASC	JRA	.406	10.666**
AHA	JRA	.288	7.737**
ASC × AHA	JRA	.141	3.792**

Model fit: $R = .604$; $R^2 = .364$; $F = 96.151$, $p < .001$.
Note. **$p < .01$.

Table S15. Conditional Indirect Effects: Anxiety-Based Pathway

Mediator	Moderator Level	Value	Effect	BootSE	LLCI	ULCI
JRA	Low AHA (-1 SD)	-1.038	-.138	.032	-.203	-.077
JRA	Mean AHA	.000	-.215	.027	-.269	-.164
JRA	High AHA (+1 SD)	1.038	-.293	.037	-.366	-.221

Table S16. Index of Moderated Mediation: Anxiety-Based Pathway

Moderator	Mediator	Index	BootSE	BootLLCI	BootULCI
AHA	JRA	-.075	.021	-.116	-.034

Table S17. Summary of Hypothesis Testing

Hypothesis	Path	Expected Direction	Result	Conclusion
H1	ASC → JRA	Positive	β = .416, p < .001	Supported
H2	JRA → PPV	Negative	β = -.593, p < .001	Supported
H3	ASC → ALM	Positive	β = .321, p < .001	Supported
H4	ALM → PPV	Positive	β = .468, p < .001	Supported
H5	AHA × ASC → JRA	Positive moderation	β = .151, p < .001	Supported
H6	ACA × ASC → ALM	Positive moderation	β = -.029, p = .340	Not supported

Table S18. PROCESS Robustness Check: Motivation-Based Pathway
Model specification: ASC → ALM → PPV, with ACA moderating the first-stage path.

**Predictor**	**Outcome: ALM**	**B**	**t**
Constant	ALM	3.673	99.429**
ASC	ALM	.309	8.372**
ACA	ALM	.416	10.837**
ASC × ACA	ALM	-.027	-.799

Model fit: R = .632; R^2 = .399; F = 111.447, p < .001.
Note. **p < .01.

Table S19. Robustness Check Using PROCESS Model 7

**Pathway**	**Moderator**	**First-stage interaction**	**Conditional indirect effect**	**Index of moderated mediation**	**Interpretation**
ASC → JRA → PPV	AHA	B = .141, t = 3.792, p < .01	Low AHA: -.138, 95% CI [-.203, -.077]; Mean AHA: -.215, 95% CI [-.269, -.164]; High AHA: -.293, 95% CI [-.366, -.221]	Index = -.075, 95% CI [-.116, -.034]	Supported
ASC → ALM → PPV	ACA	B = -.027, t = -.799, ns	Low ACA: .134, 95% CI [.087, .184]; Mean ACA: .124, 95% CI [.087, .162]; High ACA: .113, 95% CI [.076, .153]	Index = -.011, 95% CI [-.034, .012]	Not supported

Note. PROCESS Model 7 was estimated with 5,000 bootstrap samples. ASC = Ability-Based Comparison with AI with AI; JRA = job replacement anxiety; ALM = AI learning motivation; PPV = perceived professional value; AHA = AI hindrance appraisal; ACA = AI challenge appraisal. ns = non-significant.

Table S20. Conditional Indirect Effects: Motivation-Based Pathway

**Mediator**	**Moderator Level**	**Value**	**Effect**	**BootSE**	**LLCI**	**ULCI**
ALM	Low ACA (-1 SD)	-.987	.134	.025	.087	.184
ALM	Mean ACA	.000	.124	.019	.087	.162
ALM	High ACA (+1 SD)	.987	.113	.020	.076	.153

Table S21. Index of Moderated Mediation: Motivation-Based Pathway

Moderator	Mediator	Index	BootSE	BootLLCI	BootULCI
ACA	ALM	-.011	.012	-.034	.012

Table S22. Common Method Bias Diagnostics

Test	Result	Interpretation
Harman's single-factor test	First unrotated factor explained 34.43% of total variance	Below 50% threshold
Full collinearity VIF	1.660–2.175	Below 3.3 threshold

Table S23. Full Collinearity VIF Values

Construct	Full Collinearity VIF
ASC	1.988
ACA	1.660
AHA	1.661
JRA	2.061
ALM	2.060
PPV	2.175

Table S24. Effects of Control Variables on Endogenous Constructs

Control Variable	Outcome	β	t	p
Female	JRA	-.023	-.637	.524
Undisclosed gender	JRA	.046	1.272	.204
Academic level	JRA	.101	2.506	.013
Project 985 university	JRA	.017	.428	.668
Project 211 university	JRA	.037	.962	.337
Vocational/junior college	JRA	.038	1.025	.306
Other institution type	JRA	.036	.986	.325
Generative AI use frequency	JRA	.018	.435	.664
Female	ALM	.036	1.029	.304
Undisclosed gender	ALM	.033	.953	.341
Academic level	ALM	-.040	-1.015	.310
Project 985 university	ALM	.024	.620	.536
Project 211 university	ALM	.045	1.201	.230
Vocational/junior college	ALM	-.001	-.016	.987
Other institution type	ALM	.015	.430	.667
Generative AI use frequency	ALM	.180	4.294	< .001
Female	PPV	-.054	-1.607	.109

Control Variable	Outcome	β	t	p
Undisclosed gender	PPV	.070	2.073	.039
Academic level	PPV	.010	.259	.795
Project 985 university	PPV	.090	2.437	.015
Project 211 university	PPV	.070	1.937	.053
Vocational/junior college	PPV	.003	.094	.925
Other institution type	PPV	.004	.113	.910
Generative AI use frequency	PPV	.096	2.367	.018

Note. Standardized coefficients are reported. JRA = job replacement anxiety; ALM = AI learning motivation; PPV = perceived professional value. Male respondents and ordinary undergraduate institutions served as reference categories. Academic level and generative AI use frequency were treated as ordinal variables.

Table S25. Characteristics of Interview Participants

Participant	Gender	Academic level	Institution type	Field or specialization
P01	Female	First-year undergraduate	Other undergraduate institution	Network and new media
P02	Male	First-year undergraduate	Project 985 university	Journalism
P03	Female	Second-year undergraduate	Project 211 university	Communication, Sino-foreign joint program
P04	Male	Second-year undergraduate	Vocational institution	Radio and television program production
P05	Male	Third-year undergraduate	Project 985 university	Communication
P06	Female	Third-year undergraduate	Vocational institution	Online journalism and communication, Sino-foreign joint program
P07	Female	Fourth-year undergraduate	Project 211 university	Journalism, public-opinion studies
P08	Male	Fourth-year undergraduate	Other undergraduate institution	Journalism
P09	Female	First-year master's student	Project 985 university	Health communication
P10	Male	First-year master's student	Project 211 university	Political communication
P11	Female	Second-year master's student	Project 985 university	Financial communication
P12	Male	Second-year master's student	Other undergraduate institution	Health communication

Participant	Gender	Academic level	Institution type	Field or specialization
P13	Female	Third-year or later master's student	Project 211 university	International communication, Sino-foreign joint program
P14	Male	First-year doctoral student	Project 985 university	Communication and cultural industries
P15	Female	First-year doctoral student	Project 211 university	New media and society
P16	Male	Second-year doctoral student	Project 985 university	Human–AI communication
P17	Female	Second-year doctoral student	Other undergraduate institution	Political communication and online public-opinion governance
P18	Male	Third-year or later doctoral student	Project 985 university	Intelligent communication and data communication
P19	Female	Third-year or later doctoral student	Project 211 university	Health communication
P20	Male	Third-year or later doctoral student	Other undergraduate institution	Intelligent communication and national governance

Note. Participant identifiers were assigned for de-identification. Exact university names, internship organizations, and project titles are omitted to reduce the risk of deductive disclosure. "Project 985 university" and "Project 211 university" refer to the historical Chinese higher-education classifications used to distinguish nationally supported universities; the Project 211 category in this table excludes institutions also classified under Project 985. "Other undergraduate institution" refers to a degree-granting undergraduate institution outside the former Project 985 and Project 211 categories, and "Vocational institution" refers to a higher vocational college. Age is reported in grouped ranges rather than as an exact value to further protect participant confidentiality.

Table S26. Qualitative Coding Framework and Theme Development

Final Theme	Higher-Order Categories	Illustrative Initial Codes	Analytical Interpretation	Illustrative Participants
1. Human–AI Ability Comparison Was Task- and Criterion-Dep	AI task embedding and efficiency perception; human–AI	Comparison under common task materials; efficiency advantage;	Human–AI comparison was not experienced as a global judgment of whether AI or humans were generally "better." AI became a	P02, P04, P05, P06, P08, P11, P17, P19

Final Theme	Higher-Order Categories	Illustrative Initial Codes	Analytical Interpretation	Illustrative Participants
endent	capability comparison and difference attribution	task-appropriate comparison criteria; first-hand and situated experience; emotional and empathic understanding; local and cross-cultural adaptation; relational judgment; reflection on comparison fairness; shifting AI roles as tool, collaborator, or comparison target	psychologically meaningful comparison target primarily when human and AI outputs were produced under sufficiently comparable task conditions and could be evaluated using relatively explicit criteria. Participants distinguished AI advantages in speed, structure, and information processing from capabilities requiring situated knowledge, interpersonal interaction, contextual interpretation, and responsibility.	
2. Task-Level Capability Differences Were Linked to Replacement Anxiety through Occupational Interpretation	Efficiency-related capability gaps; emotional and occupational reappraisal; risk and responsibility boundaries	Efficiency shock; job replacement anxiety; perceived compression of routine work; increasing demands for composite skills; consequence-based task differentiation; high-responsibility judgment as non-delegable; anxiety–reflection–action transition; re-evaluation of standardized entry-level work	A task-level AI advantage was not automatically interpreted as occupational replacement. Anxiety became more salient when participants generalized AI's efficiency advantage from particular tasks to declining demand for their own skills, reduced security of entry-level work, or uncertainty about future professional requirements. Conversely, anxiety was less generalized when participants located AI's advantage primarily in standardized tasks and retained meaningful human responsibility for consequential judgments.	P01, P02, P05, P08, P11, P17, P19, P20
3. Comparison Was Linked to	Task-driven adaptive	Efficiency released as an	Participants often used comparison diagnostically.	P01, P02, P05, P06,

Final Theme	Higher-Order Categories	Illustrative Initial Codes	Analytical Interpretation	Illustrative Participants
Learning Motivation through Diagnosed Gaps and Concrete Task Demands	action; workflow adjustment and capability development	opportunity for higher-value work; task-triggered learning; small-goal experimentation; workflow integration; auditable prompt design; version and decision logs; human cross-checking; counterexample and alternative-explanation testing; practice-based training; development of data and methodological literacy; course and supervisor requirements	Rather than responding to an abstract belief that "AI is important," they identified specific and actionable capability gaps through coursework, internships, research tasks, feedback, deadlines, or professional requirements. These concrete demands translated discrepancies into learning objectives and workflow adjustments, helping explain why comparison could remain associated with AI learning motivation even when challenge appraisal did not significantly amplify the relationship.	P11, P16, P17, P18, P19, P20
4. Comparison with AI Was Associated with a More Specific Understanding of Professional Value	Risk and responsibility boundaries; responsibility-oriented human–AI collaboration; professional value repositioning	Source and original-material verification; contextual qualification checking; protection of sensitive information; first-hand reporting and field experience; emotional and audience understanding;	Comparison with AI prompted some participants to reconsider professional value in more differentiated terms. Rather than locating professional value primarily in the speed or fluency of content production, participants emphasized evidence verification, contextual understanding, field engagement, interpersonal relationships, ethical judgment, public accountability, and responsibility for consequential decisions. AI was therefore	P02, P05, P06, P10, P11, P12, P16, P17, P19, P20

Final Theme	Higher-Order Categories	Illustrative Initial Codes	Analytical Interpretation	Illustrative Participants
		cross-cultural adaptation; public consequences and ethical responsibility; uncertainty disclosure; professional-value repositioning; organizational responsibility chains; human–AI capability recombination	increasingly understood as part of a reconfigured professional workflow rather than as a complete substitute for professional competence.	

Figure S1. Structural Model with Standardized Path Coefficients

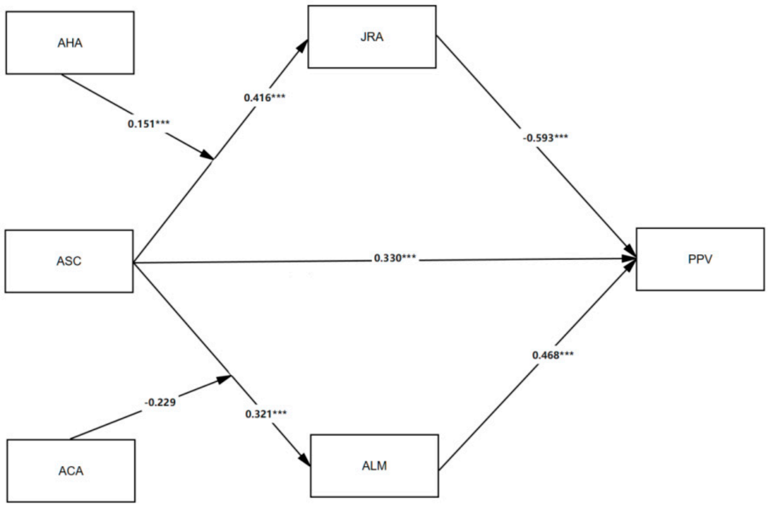

Note: Dashed paths indicate moderation effects. *p < .05, **p < .01, ***p < .001.
